# Supplementary material for: Cervical pessary versus vaginal progesterone in women with a multiple pregnancy and a short cervix: A randomised controlled trial
Source: PLoS Med. 2025 Nov 3;22(11):e1004586. doi: 10.1371/journal.pmed.1004586 (PMC12591417; doi:10.1371/journal.pmed.1004586)
Supplement: S1 File — Table A: Protocol amendments and deviations. Table B: Details of intervention use: reasons for premature termination of allocated intervention on maternal level. Table C: Per protocol analyses for various compliance thresholds of proportion of days covered (PDC) on child level. Table D: Serious Adverse Events. Table E: Exploratory analysis for cervical length ≤25 mm and >25 mm on the secondary outcomes (s)PTB < 37, (s)PTB < 34, (s)PTB < 32, (s)PTB < 28 and (s)PTB < 24 weeks. (PDF) [file pmed.1004586.s001.pdf]

A. S1 Supporting tables Quadruple P multiples study

Protocol amendments	Details	Date of approval by authority (MEC)
1.2	Minor textuality revisions: Besins Healthcare selected as drug supplier; added drug accountability documentation.	18-10-2013
1.3	Clarified standard treatment offered to non-participants.	21-12-2013
1.4	PI changed from B.W. Mol to E. Pajkrt; inclusion window for singletons extended to 18–22 weeks conform structural anomaly scan; updated sample size.	22-08-2014
2.0	Inclusion window for multiples also extended to 22weeks conform singletons plus change from twins in multiples (including triplets and quadruplets); removed placenta praevia as exclusion; updated sample size and sites; clarified SAE definitions; Clarified sections by removing repetitive text, better distinction between singleton and multiple pregnancies, and specified use of vaginal progesterone capsules.	08-06-2016
2.1	Clarified cervical length measurement and consent procedures in non-routine centres.	08-06-2016
2.2	Removed term 'observational cohort'; separate protocol for screening study initiated.	08-06-2016
2.3	Added missing component (Retinopathy Of Prematurity) to composite outcome; aligned variables with PROMPT guidelines.	06-10-2016
2.4	Revised SAE reporting based on DSMC input and safety interim analysis at 1/3 and 2/3 of inclusions; clarified 'multiple' vs. 'twin' terminology; refined exclusion criteria PTB <34 weeks GA into spontaneous PTB <34 weeks of gestation, which was the intention.	19-04-2017
2.5	Script of counseling movie; update PIF	4-12-2017
2.6	Progress report to the MEC. Change in study end date and updates to the site-specific monitoring plan.	12-03-2018
2.7	Updated list of local PIs; removed outdated protocol sections; because on the 25th of May 2018, The Dutch Data Protection Act (WBP) has been replaced by the GDPR, necessary replacements are made in accordance with the GDPR; clarified AE/SAE definitions; Added an appendix to informed consent forms to collect contact details for future research contact and an option to allow or decline use of data for future studies.	29-10-2018
2.8	Updated list of PIs; updated English Informed Consent information.	12-12-2018
2.9	Re-added medication diary; removed inactive sites from protocol; study medication (vaginal progesterone) now provided as standard care via participants' own pharmacies, in line with new national guideline; clarified that inclusion involves asymptomatic cervical shortening (was explained in text but not specified in exclusion criteria's); added consent procedure for observational cohort to protocol (already included in patient information); added collection of race and ethnicity to patient materials, based on scientific rationale and ethical review.	13-07-2020

Protocol Deviations	Details
Individual participant level	
Switched treatment	7 participants switched to other treatment modality (5 from pessary to progesterone, 2 from progesterone to pessary)
Exclusion of participants	- In hindsight not eligible (N=5)

	- ICF not available and not well enough documented that inclusion went according to protocol (N = 7)<br>- Withdrawn and no consent to safe data (N=2)
Informed consent procedure not according to protocol	63 participants informed consent procedure not completely according to protocol
Study Level	
The initial protocol and early amendments were not submitted to the competent authority (CCMO).	
The accountability log for the medical device (pessary) was not available.	
Adverse events and serious adverse events were reported late to the MEC.	
Medical diaries were either not returned, no longer in the possession of participants, or never provided.	
Prior to Amendment 2.9, the original supplier of the study medication (Besins) had discontinued delivery, so prescriptions were issued for regular medication from the same manufacturer.	
Until April 2019, only line listings of SAEs and AEs were submitted to the authorities, without an accompanying safety progress report.	
Until 2018, it was unclear which adverse events and serious adverse events were required to be reported.	

SAE, serious adverse event; AE, adverse event; PROMPT, Prospective Meta-analysis for Pessary Trials Study Protocol; MEC, Medical Ethics Committee; DSMC, data safety monitory committee; PTB, preterm birth; PIF, patient information form (including informed consent form); GDPR, General Data Protection Regulation; PI, principal investigator;

Table A – Protocol amendments and deviations

	Pessary (n = 133)		Progesterone (n = 129)	
Allocated treatment not initiated	4 (3.0%)		3 (2.3%)	
Pessary placement failure	2 (50.0%)		NA	
Patient preference	2 (50.0%)		3 (100.0%)	
Removal of pessary or stop progesterone	129 (97.0%)		126 (97.7%)	
*According to study protocol^*	*76 (58.9%)*		*85 (67.5%)*	
Gestational age > 36 weeks	37 (48.6%)		38 (44.7%)	
Contractions or labour	19 (25.0%)		24 (19.0%)	
Other reasons, required delivery	10 (13.2%)		7 (5.6%)	
(P)PROM	10 (13.2%)		16 (12.7%)	
*Not according to study protocol^*	*53 (41.1%)*		*41 (32.5%)*	
	Stopped 48 (90.6%)	*Switched 5 (9.4%)*	*Stopped 39 (95.1%)*	*Switched 2 (4.9%)*
Discomfort and/or excessive discharge	6 (12.5%)	2 (40%)	5 (12.8%)	1 (50%)
Patient preference	0 (0%)	2 (40%)	0 (0%)	1 (50%)
Pessary replacement failure	4 (8.3%)	1 (10%)	NA	NA
Vaginal blood loss	5 (10.4%)	0 (0%)	1 (2.6%)	0 (0%)
Abdominal pain/cramps	14 (29.2%)	0 (0%)	10 (25.6%)	0 (0%)
PCD <100%	7 (14.6%)	NA	9 (23.1%)	NA
Other	10 (20.8%)	0 (0%)	11 (28.2%)	0 (0%)
Cerclage	2 (4.2%)	0 (0%)	3 (7.7%)	0 (0%)

^ If multiple reasons were given, one was chosen to be displayed in table
PDC, proportion of days covered; PPROM, premature preterm rupture of membranes

Table B – Details of intervention use: reasons for premature termination of allocated intervention on maternal level

	Compliance threshold (% PDC)	Total N	Pessary n/N (%)	Progesterone n/N (%)	RR /mean difference (95% CI)	p-value
Composite adverse neonatal outcome, crude	100%	327	30/154 (19.5%)	23/173 (13.3%)	1.47 [0.74, 2.91]	0.28
	90%	400	31/193 (16.1%)	23/207 (11.1%)	1.45 [0.73, 2.88]	0.30
	80%	422	37/213 (17.4%)	23/209 (11.0%)	1.58 [0.82, 3.06]	0.18
	70%	436	37/217 (17.1%)	25/219 (11.4%)	1.49 [0.78, 2.85]	0.22
	60%	446	43/225 (19.1%)	27/221 (12.2%)	1.56 [0.85, 2.88	0.15

PDC, proportion of days covered

Table C - Per protocol analyses for various compliance thresholds of PDC on child level

Serious Adverse Events	Pessary *(n=133)*	Progesterone *(n=129)*	Relative Risk (95% CI)	p-value
Maternal death	0 (0%)	0 (0%)	N.A.	N.A.
Life threatening (at the time of event) to the mother	2 (1.5%)	0 (0%)	N.A.	N.A.
Hospitalization or prolongation for other than expected reason#	3 (2.3%)	1 (0.8%)	2.91 [0.31, 27.6]	0.35
Persistent or significant disability or incapacity of the mother	0 (0%)	0 (0%)	N.A.	N.A.
Severe congenital anomaly or birth defect of the neonate	0 (0%)	0 (0%)	N.A.	N.A.
Any other important medical event$	0 (0%)	0 (0%)	N.A.	N.A.
Total	5 (3.8%)	1 (0.8%)	4.85 [0.57, 40,9]	0.15

#Requires hospitalization or prolongation of existing inpatients' hospitalization other than expected obstetric complications (such as threatened premature labour, admissions due to spontaneous or indicated labour.
$Any other important medical event that may not result in death, be life threatening, or require hospitalization, may be considered a serious adverse experience when, based upon appropriate medical judgement, the event may jeopardize the subject or may require an intervention to prevent one of the outcomes listed above.

Table D– Serious Adverse Events

	Pessary N = 133	Progesterone N = 129
**Cervical length ≤25mm**	N = 30	N = 24
PTB < 37 weeks	25 (83.3%)	21 (87.5%)
sPTB < 37 weeks	22 (73.3%)	16 (66.7%)
PTB < 34 weeks	18 (60.0%)	13 (54.2%)
sPTB < 34 weeks	18 (60.0%)	13 (54.2%)
PTB < 32 weeks	15 (50.0%)	12 (50.0%)
sPTB < 32 weeks	15 (50.0%)	12 (50.0%)
PTB < 28 weeks	9 (30.0%)	8 (33.3%)
sPTB < 28 weeks	9 (30.0%)	8 (33.3%)
PTB < 24 weeks	4 (13.3%)	5 (20.8%)
sPTB < 24 weeks	4 (13.3%)	5 (20.8%)
**Cervical length >25 mm**	N = 103	N = 105
PTB < 37 weeks	70 (68.0%)	74 (70.5%)
sPTB < 37 weeks	38 (36.9%)	44 (41.9%)

PTB < 34 weeks	29 (28.2%)	25 (23.8%)
sPTB < 34 weeks	19 (18.4%)	22 (21.0%)
PTB < 32 weeks	15 (14.6%)	15 (14.35)
sPTB < 32 weeks	13 (12.6%)	13 (12.4%)
PTB < 28 weeks	11 (10.7%)	7 (6.7%)
sPTB < 28 weeks	11 (10.7%)	6 (5.7%)
PTB < 24 weeks	4 (3.9%)	1 (1.0%)
sPTB < 24 weeks	4 (3.9%)	1 (1.0%)

PTB, preterm birth; sPTB, spontaneous preterm birth; N, number

Table E – Exploratory analysis for cervical length ≤25mm and >25mm on the secondary outcomes (s)PTB <37, (s)PTB < 34, (s)PTB <32, (s)PTB <28 and (s)PTB < 24 weeks
