# Supplementary material for: Cervical pessary versus vaginal progesterone in women with a multiple pregnancy and a short cervix: A randomised controlled trial
Source: PLoS Med. 2025 Nov 3;22(11):e1004586. doi: 10.1371/journal.pmed.1004586 (PMC12591417; doi:10.1371/journal.pmed.1004586)
Supplement: S2 File — (PDF) [file pmed.1004586.s002.pdf]

Pessary or Progesterone to Prevent Preterm delivery in women with short cervical length

-Quadruple P trial-

PROTOCOL TITLE: 'Pessary or Progesterone to Prevent Preterm delivery in women with a short cervical length'

**Protocol ID**	Quadruple P
**Short title**	Pessary or Progesterone to Prevent Preterm delivery in women with short cervical length
**Coordinating investigator/project leader**	Prof. dr. E. Pajkrt, gynecologist<br>[redacted]<br>Postbus 22660<br>1100 DD Amsterdam Zuidoost
**Principal investigator**	Prof. dr. E. Pajkrt, gynecologist<br>[redacted]<br>Postbus 22660<br>1100 DD Amsterdam Zuidoost
**Investigator per site**	***For investigator per site see list of participants sites.***
**Sponsor (in Dutch: verrichter/opdrachtgever)**	Amsterdam Universtity Medical Center – location AMC
**Independent expert:**	[redacted], gynaecologist, AMC
**Pharmacy:**	IMP is part of standard care.

PROTOCOL SIGNATURE SHEET

**Name**	**Signature**	**Date**
**[Coordinating Investigator/Project leader/Principal Investigator]:**<br>**Prof. dr. E. Pajkrt, gynaecologist**		

TABLE OF CONTENTS

1. STUDY DESIGN 11
2. STUDY POPULATION 12
2.1 Population (base) 12
2.2 Inclusion criteria 13
2.3 Exclusion criteria 13
2.4 Sample size calculation 13
3. TREATMENT OF SUBJECTS 15
3.1 Investigational product/treatment 15
4. INVESTIGATIONAL PRODUCT 16
4.1 Name and description of investigational product(s) 16
4.2 Summary of findings from non-clinical studies 16
4.3 Summary of findings from clinical studies 16
4.4 Summary of known and potential risks and benefits 17
4.5 Description and justification of route of administration and dosage 17
4.6 Dosages, dosage modifications and method of administration 17
4.7 Preparation and labelling of Investigational Medicinal Product 18
4.8 Drug accountability 18
5. METHODS ..... **Fout! Bladwijzer niet gedefinieerd.**
5.1 Study parameters/endpoints 18
5.1.1 Main study parameter/endpoint 18
5.1.2 Secondary study parameters/endpoints (if applicable) 18
5.1.3 Long term follow-up 19
5.2 Randomisation, blinding and treatment allocation 19
5.3 Study procedures 19
5.4 Withdrawal of individual subjects 20
5.5 Premature termination of the study 20
6. SAFETY REPORTING
6.1 Temporary halt for reasons of subject safety 28
6.2 AEs, SAEs and SUSARs 28
6.2.1 Adverse events (AEs) 28
6.2.2 Serious adverse events (SAEs) 29
6.3 Follow-up of adverse events 31
6.4 Data Safety Monitoring Board (DSMB) 32
7. STATISTICAL ANALYSIS 32
7.1 Primary study parameter(s) 232
7.2 Secondary study parameter(s) 23
7.3 Other study parameters 23
7.4 Interim analysis (if applicable) 24
7.5 Regulation statement 25

7.6 Recruitment and consent 25
7. Compensation for injury 25
8. ADMINISTRATIVE ASPECTS, MONITORING AND PUBLICATION 25
8.1 Handling and storage of data and documents 25
8.2 Amendments 26
8.3 Annual progress report 26
8.4 End of study report 26
8.5 Public disclosure and publication policy 26
9. STRUCTURED RISK ANALYSIS 27
90.1 Potential issues of concern 27
9.2 Synthesis 29
10. REFERENCES 30

LIST OF ABBREVIATIONS AND RELEVANT DEFINITIONS

ABR	ABR form, General Assessment and Registration form, is the application form that is required for submission to the accredited Ethics Committee (In Dutch, ABR = Algemene Beoordeling en Registratie)
AE	Adverse Event
AR	Adverse Reaction
CA	Competent Authority
CCMO	Central Committee on Research Involving Human Subjects; in Dutch: Centrale Commissie Mensgebonden Onderzoek
CV	Curriculum Vitae
DSMB	Data Safety Monitoring Board
EU	European Union
EudraCT	European drug regulatory affairs Clinical Trials
GCP	Good Clinical Practice
IB	Investigator's Brochure
IC	Informed Consent
IMP	Investigational Medicinal Product
IMPD	Investigational Medicinal Product Dossier
METC	Medical research ethics committee (MREC); in Dutch: medisch ethische toetsing commissie (METC)
(S)AE	(Serious) Adverse Event
SPC	Summary of Product Characteristics (in Dutch: officiële productinfomatie IB1-tekst)
Sponsor	The sponsor is the party that commissions the organisation or performance of the research, for example a pharmaceutical company, academic hospital, scientific organisation or investigator. A party that provides funding for a study but does not commission it is not regarded as the sponsor, but referred to as a subsidising party.
SUSAR	Suspected Unexpected Serious Adverse Reaction
GDPR/AVG	General Data Protection Regulation (in Dutch: Algemene Verordering Gegevensbescherming)
WMO	Medical Research Involving Human Subjects Act (in Dutch: Wet Medisch-wetenschappelijk Onderzoek met Mensen

SUMMARY

Rationale: Preterm birth (PTB) is in quantity and in severity the most important issue in obstetric care in the developed world. Progestagens and cervical pessaries are both considered as potential preventive treatments.

Objective: We aim to compare the effectiveness of vaginal progesterone and cervical pessary in the prevention of preterm birth in women with an asymptomatic short cervix, in singleton and multiple pregnancies separately.

Study design: Nationwide open-label multicentre randomized clinical trial with an economic analysis alongside it.

Study population: Women with a singleton or multiple pregnancy undergoing fetal assessment (at 18-22 weeks for singleton pregnancy and 16-22 weeks for multiple pregnancies) will be offered cervical length measurement. Women with an asymptomatic short cervix (singleton pregnancy 35 mm or less (11.5th percentile), multiple pregnancy less than 38 (25th percentile), will be invited to participate in the study.

Intervention (if applicable): Eligible woman will be randomly allocated to receive either a pessary or progesterone. In case of progesterone, 200 mg of progesterone will be daily self-administered vaginally following randomization. In case of a silicone cervical pessary, this will be placed between 18-22 weeks for singletons or between 16-22 weeks for multiples by a simple vaginal examination. Both interventions will be applied until 36 weeks gestation or until delivery, whatever comes first.

Main study parameters/endpoints: Primary outcome will be composite adverse perinatal outcome including both morbidity and mortality , specifically: severe Respiratory Distress Syndrome (RDS), Bronchopulmonary Dysplasia (BPD), Intraventricular Haemorrhage grade III and IV (IVH), Periventricular Leucomalacia (PVL) higher then grade I, Necrotizing Enterocolitis (NEC), Retinopathy of Prematurity (ROP) higher then stage I, culture proven sepsis, (intrapartum) stillbirth and death before discharge from the nursery, all measured up until 10 weeks after the expected term date. Secondary outcomes will be time to delivery, preterm birth rate before 28, 32, 34 and 37 weeks, (days of) admission in neonatal intensive care unit, patent ductus arteriosus (PDA), treated seizures, premature rupture of the membranes (PPROM), tocolysis (duration), use of corticosteroids, use of magnesium sulphate, mode of delivery, Twin Transfusion Syndrome (TTS), maternal morbidity, maternal admission days for preterm labour and costs.

Nature and extent of the burden and risks associated with participation, benefit and group relatedness:

BURDEN

Eligible women, i.e. those with a cervical length less than or equal to 35 mm for singleton pregnancies measured between a gestational age of 18 to 22, and less than 38 mm for multiple pregnancies measured between 16 to 22 weeks' gestation, will be randomly allocated to receive either a cervical pessary or vaginal progesterone. Those randomised to progesterone will be given Utrogestan 200mg, one vaginal capsule per day, for the amount of days left till 36 weeks of gestation. Capsules will be taken

at time of randomisation up to 36 weeks gestational age or delivery, whatever comes first. The capsules will be self-administered vaginally by patients on a daily basis.

A cervical pessary will be placed in situ between 18-22 weeks for singletons and between 16-22 weeks for multiples. The pessary is made of silicone, which is soft and flexible. It is folded and put around the cervix by a simple vaginal examination without causing any pain. The pessary will remain within the vagina during pregnancy, until 36 weeks or until delivery, whatever comes first. In a questionnaire evaluation, Arabin et al. found a generally positive opinion of the pessary treatment (*Arabin et al., 2003*). 75% of that group would use the pessary again and would even recommend it to others. They reported that there might be an increase of vaginal discharge. If there is an increase in discharge, women will be evaluated for vaginal infection and treated if necessary.

Previous studies showed no fetal adverse effects after pessary placement.

BENEFIT

Since patients with a short cervical length are at increased risk of preterm delivery, they will benefit, as a pessary and vaginal progesterone are considered effective in reducing preterm birth in these women. The aim of this study is to find out which treatment modality is more effective in singleton and in multiple pregnancies.

INTRODUCTION AND RATIONALE

Preterm birth (PTB) is defined as delivery before 37 completed weeks of gestation. PTB affects 12,000 pregnancies per year in the Netherlands and is the most important cause of neonatal mortality and morbidity, and subsequent neurodevelopmental sequelae. Of all perinatal mortality, 50% to 70% can be attributed to preterm birth, with increased mortality rates for those being delivered more prematurely. Prior to 25 weeks, the chance of survival is approximately 60%. At 28 and 32 weeks, survival rates are 75% and 95%, respectively. Similarly, neonatal morbidity is strongly increased in case of preterm birth. A follow up study among a cohort of Dutch children born prior to 32 weeks showed that the rate of a severe handicap among surviving children was 10% (*Walther et al., 2000*).

In women with a singleton pregnancy, the spontaneous preterm birth rate in The Netherlands is just under 5%, resulting in a perinatal mortality rate of 0.8% and a severe disability rate of 0.7%, while 2% of the children suffer from moderately disability (*Schaaf et al., 2011*). Women with a twin pregnancy have a much higher risk of preterm delivery. The incidence of preterm delivery in twin pregnancies is almost 50%, with 1.8%, 7% and 14% delivering before 28, 32 and 34 weeks of gestation, respectively. These results among women with a twin pregnancy in 8% perinatal mortality, 7% severely disabled children and 20% moderately disabled children (*Lim et al., 2007*).

The costs of preterm delivery are enormous. These costs are mainly due to intensive care for neonates at a cost of € 1,500 per day (with an average duration of admission of 28 days). Estimated costs of disabled children are € 80,000 yearly for severely and € 20,000 for moderately handicapped children. Additionally, substantial costs result from antenatal care for mothers at increased risk for preterm delivery.

Until a decade ago, there were no interventions for the prevention of preterm birth. However, in the last decade two important breakthroughs have been established. First, two randomized clinical trials demonstrated that progestagens reduced preterm birth in women with a previous preterm birth with 50% (*Da Fonseca et al., 2003; Meis et al., 2003*). In 2010, a Cochrane systematic review confirmed this effectiveness of progesterone in the prevention of preterm birth (risk ratio of 0.33; 95% CI 0.17-0.67)(*Su et al., 2010*). Moreover, a recent meta-analysis of individual patient data (*Romero et al., 2012*) indicated that in women with a short cervix, who are known to have an increased risk for preterm birth, progesterone generated lower rates of preterm delivery (26% vs. 36%; OR 0.45, 95% CI 0.25-0.80) and lower rates of perinatal mortality, although this difference did not reach statistical significance (15% vs. 17%; OR 0.69, 95% CI 0.38-1.3).
An individual patient data (7536 children) meta-analysis of our group on thirteen trials in multiple pregnancies using progestagens(intramuscular 17-hydroxyprogesterone caproate (17Pc) or vaginal progesterone) shows no overall effect of progestagens on adverse perinatal outcome in twin pregnancies (RR 1.1; 95% CI 0.97-1.4 for 17Pc and RR 0.97; 95% CI 0.77-1.2 for vaginal progesterone) (*Schuit et al., 2012*). However, vaginal progesterone showed to reduce adverse perinatal outcome among women with a cervical length equal or below 25mm measured at randomisation or before 24 weeks of gestation (15/56 vs. 22/60; RR 0.57; 95% CI 0.47-0.70 and 14/52 vs. 21/56; RR 0.56; 95% CI 0.42-0.75, respectively).
A second potential breakthrough in the prevention of preterm birth is the use of a cervical pessary. The PECEP trial (*Goya et al., 2012*) showed that cervical pessary, in women with a singleton pregnancy and short cervical length (<25 mm), reduced preterm birth before 34 weeks (6% versus 27%). Within the Dutch obstetric research consortium, we finished the ProTwin trial (projectnr:50-50110-96-618) which studied the effectiveness of pessary in unselected twin pregnancies. Although there was no overall treatment effect of the pessary, the pessary significantly reduced preterm delivery rates in a pre-specified subgroup of women with a cervix less than the 25th percentile (38 mm) (11% vs. 25%, RR 0.44; 95%CI 0.20-0.98 for delivery less than 32 weeks). More importantly, adverse perinatal outcome rate was substantially lower in the pessary group as compared to no intervention (7% vs. 30%, RR 0.23 95% CI; 0.09-0.60).

In view of the studies mentioned above, two conclusions can be drawn. First, a short cervix measured at the second trimester identifies women at risk for preterm birth. Second, both progestagens and cervical pessary are promising treatments for women who are at increased risk for preterm birth due to a short cervix. Up to now, no study has compared treatment with progesterone and cervical pessary. In view of the need for additional data on the relative effectiveness of both treatment modalities, we propose an randomized controlled trial in which cervical length will be measured in women with singleton (GA 18-22 weeks) and multiple (GA 16-22 weeks) pregnancies. Subsequently we will randomize women with a short cervix, ≤ 35 mm in singletons and < 38 mm in multiples, to cervical pessary or vaginal progesterone. The data will be used to assess which of both treatments is best in the prevention of adverse perinatal outcome resulting from preterm delivery, and to assess the cost-effectiveness as well

as budget impact from screening programs using cervical length in women with singleton and in women with a twin pregnancy.

RELEVANCE FOR CLINICAL PRACTICE

The relevance of the proposed study is in our opinion beyond doubt. As stated earlier, spontaneous preterm delivery is the single most important cause of perinatal mortality in the western World, and prevention of preterm birth is a major goal of obstetrical care. Ultrasonographic measurement of cervical length at 20-23 weeks of gestation identifies women at risk for preterm birth (*Iams et al., 1996*). During pregnancy, most women do not give serious consideration to the possibility of preterm delivery. In most cases, admission of an infant to the neonatal unit is unexpected and is stressful for the parents (*Fowlie et al., 2004*). Until recently, preventive treatments were not available. In view of the potential of vaginal progesterone and cervical pessary as described above, these interventions are likely to be introduced in clinical practice. The present project is needed to guide the choice between pessary and progesterone, thus reducing potential future practice variation.

IMPLEMENTATION FEASIBILITY

The study involves all levels of care in the Netherlands, from midwifery practises to academic hospitals. This offers a unique possibility for nationwide implementation of the knowledge that will become available from this project. Thanks to the infrastructure already established in the Triple P trial (project number MEC_AMC_08/374) (a multicentre study to evaluate whether a screening program with cervical length measurement to find women at risk for a preterm delivery, is cost-effective), we will be able to start with this project soon after finishing the Triple P study, thus enhancing efficacy and decreasing costs. Furthermore, it will enhance collaboration between the levels of care, which will in turn lead to increased quality of care due to increased referral rates of women at risk and the possibility of quality control.

Besides, all levels of care will be familiar with the study protocol and the results of the study, which will facilitate implementation (*Litjens et al., accepted 2012*). Previously, we already demonstrated that studies performed in our nationwide consortium lead to implementation even before they are published in scientific literature or implemented in clinical guidelines (*van der Tuuk et al., 2011*).

The results of the study will be incorporated in the national guidelines of KNOV and NVOG soon after publication. Obstetrical guidelines are a national reference for local protocols, and midwives and gynaecologists are likely to follow the guidelines based on the result of the study. In addition, the results of the study will be presented on international conferences and published in international peer-reviewed journals.

OBJECTIVES

Primary Objective:

Primary outcome is composite adverse perinatal outcome. This composite outcome contains periventricular leukomalacia (PVL) > grade 1, severe respiratory distress syndrome (RDS), bronchopulmonary Dysplasia (BPD), Intraventricular Haemorrhage (IVH) grade III or IV, Necrotizing

Enterocolitis (NEC) > stage 1, Retinopathy of prematurity (ROP), proven sepsis, (intrapartum) stillbirth and death before discharge from the nursery, all measured up until 10 weeks after the expected due date.

Secondary Objectives:
Secondary outcome measures are time to delivery, preterm birth rate before 28, 32, 34 and 37 weeks (spontaneous, iatrogenic and total), birth weight, (days of) admission in neonatal intensive care unit, patent ductus arteriosus (PDA), treated seizures, premature rupture of the membranes (PPROM), tocolysis (duration), use of corticosteroids, use of magnesium sulphate, mode of delivery, Twin Transfusion Syndrome (TTS), maternal morbidity (specifically, thromboembolic complications, infections, pneumonia, endometritis, eclampsia/HELLP or death) and maternal admission days for preterm labour. A cost-effectiveness analysis will be performed. This will be measured until 10 weeks after the expected term date.
All components of the composite outcome will also be assessed separately as secondary outcome. All of the core-outcomes for research concerning interventions to prevent preterm birth as defined by the CROWN-initiative (*van t Hooft et al 2016*) will be assessed.

1. STUDY DESIGN

Study design:

Multicenter randomized controlled study. It is a superiority trial comparing the effectiveness of vaginal progesterone and pessary in the reduction of adverse perinatal outcomes. We will investigate women with a singleton and women with a multiple pregnancy separately. For singleton pregnancies, the standard intervention is vaginal progesterone, so pessary will be compared with vaginal progesterone, while standard intervention is a pessary for multiple pregnancies, so in multiples we will compare vaginal progesterone with a pessary.

Setting:

Measuring cervical length at 20 weeks' gestation is common practice in the participating hospitals, which makes execution of this study in these groups relatively simple.

a. singleton pregnancy

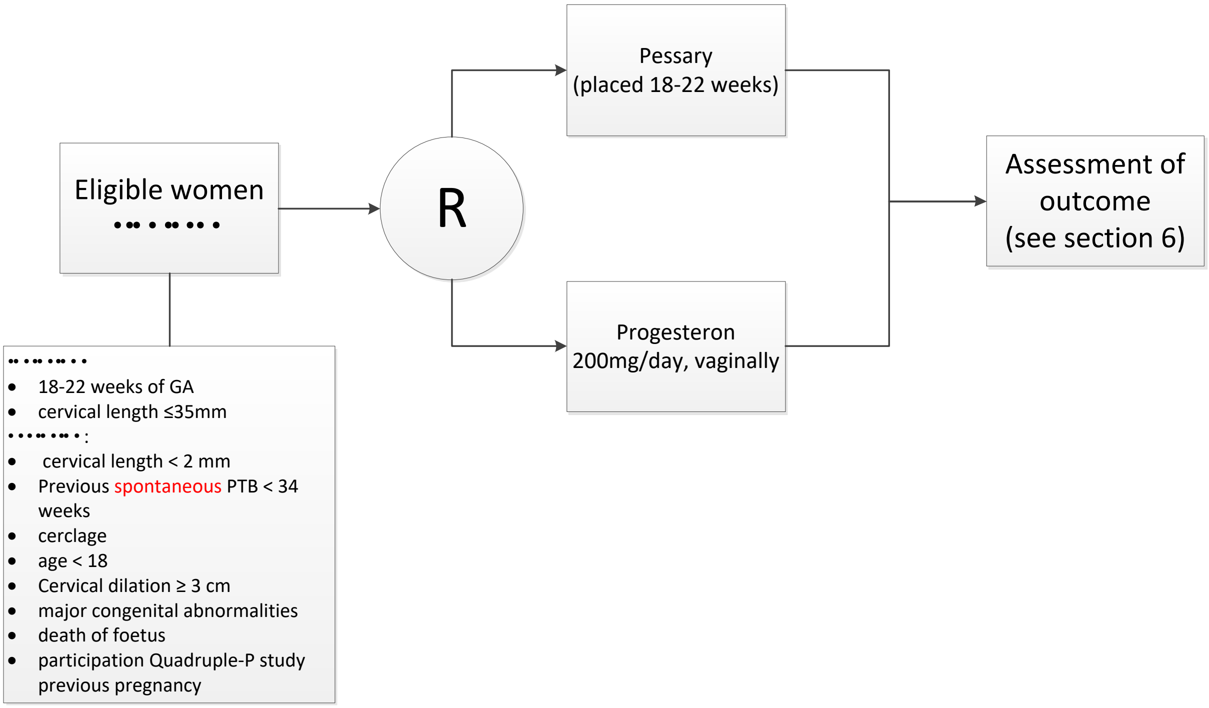

b. multiple pregnancy

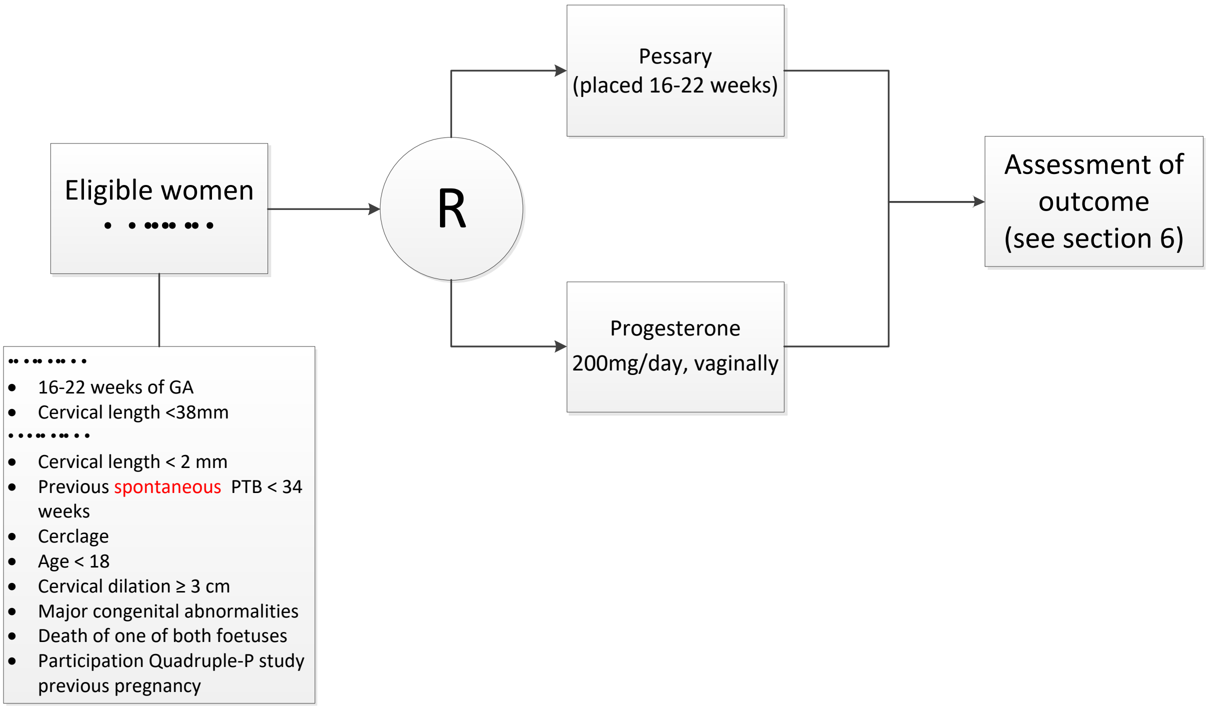

2. STUDY POPULATION

2.1 Population (base)

At present, all pregnant women are offered a structural fetal assessment ultrasound around 20 weeks of gestation. In the present study, we will offer pregnant women with a singleton (between 18 and 22 weeks of gestation) or multiple pregnancy (between 16 and 22 weeks of gestation) the possibility to screen for short cervix to identify an increased risk of preterm delivery. Women with singleton pregnancies receive their antenatal care in a midwifery practice or hospital. Their cervical lengths will be measured during the routine ultrasound at 20 weeks of gestation. In most hospitals and ultrasound practices participating in the Quadruple P study, this cervical length measurement is common practice. Women with multiples pregnancies will receive their antenatal check-ups at the hospital. In most hospitals, it is common practice to measure cervical length in multiples, either routinely or in the context of studies, such as the AMPHIA or ProTWIN study. In some centres cervical length measurement is not routinely performed. Women who receive antenatal care in these centres will be asked to give informed consent prior to this procedure.

[*Lim et al., 2011, Liem et al. 2013*]. In all women, cervical length is measured prior to randomisation. The women will be asked to empty their bladder prior to the structural ultra-sonographic foetal assessment. Transvaginal ultrasonography will be carried out with a 5-MHz transducer. The probe will be placed in the anterior fornix of the vagina and a sagittal view of the cervix, with the echogenic endocervical mucosa along the length of the canal, will be obtained. The callipers will be used to measure the distance between the triangular area of echodensity at the external os and the V-shaped notch at the internal os. Each examination will be performed during a period of about three minutes to observe any cervical changes that may arise due to contractions. In such cases, the shortest measurement will be recorded. The presence or absence of funnelling at the internal os will also be

recorded. If the cervical length of the pregnant woman is below the 11.5th percentile (≤35 mm) in singletons and below the 25th percentile (<38 mm) in multiples and she is asymptomatic (no blood loss or contractions etc.), she will be asked to participate in the treatment part of the trial in which we compare vaginal progesterone and pessary. Women will be asked to give informed consent prior to participation in the study.

Women who are eligible for the study but who do not give informed consent, are registered and outcome data will be collected, unless patients have withdrawn consent to use their medical records for research purposes. In consultation with the treating gynaecologist, women who declined participation in the trial will be offered treatment according to local protocol.

2.2 Inclusion criteria

In order to be eligible to participate in this study, a subject must meet either the first or the second criteria:

1. Singleton pregnancy and cervical length at 18 to 22 weeks of 35 mm or less (≤35mm)
2. Multiple pregnancy and cervical length at 16 to 22 weeks of less than 38 mm (<38mm)
3. Written informed consent

2.3 Exclusion criteria

A potential subject who meets any of the following criteria will be excluded from participation in this study:

1. Cervical cerclage in this pregnancy
2. Maternal age less than 18 years
3. Identified major congenital abnormalities in this pregnancy
4. Death of one or both of the foetuses in this pregnancy
5. Spontaneous preterm birth < 34 weeks in previous pregnancies
6. Participation Quadruple P study in previous pregnancies
7. Cervical length < 2mm
8. Cervical dilatation ≥ 3cm
9. Signs of blood loss or contractions

2.4 Sample size calculation

In singleton pregnancies, we expect a reduction of adverse neonatal outcome from 5% in the vaginal progesterone group to 1% in the pessary group. The adverse neonatal outcome of 5% in the vaginal progesterone is based on the Dutch TripleP study which was performed in a comparable population, The risk reduction to 1% in the pessary group is based on the PECEP trial were a 3% reduction of poor neonatal outcome was found. However, in this study only women with a cervix below 25 mm were eligible. Since we include women with a cervix below 35 mm, we expect a lower adverse neonatal outcome in our study population. Using a two-sided test with a type I error of 5% and type II error of 20%

and a loss to follow-up of 10%, we calculated that we would need a sample size of 628women (314 per group).
For multiple pregnancies, we expected frequencies of adverse perinatal outcome of 24% per pregnancy in the vaginal progesterone group and 12 % in the pessary group. In the ProTwin trial adverse neonatal outcome was 12% in a comparable population with a similar cervical length. In the IPD meta-analysis of Schuit et al. adverse outcome was 25% in a population with a shorter cervix (< 25 mm). We expect a 50% reduction of adverse neonatal outcome in the pessary group. Since twin gestations are followed up in the hospital, we expect less patients being lost to follow up. Using a two-sided test with a type I error of 5% and type II error of 20% and a loss to follow-up of 5%, we calculated we would need a sample size of 332 women (166 per group).

Singleton pregnancies: With our previous experience in the Triple P trial, we know that screening 850 low risk women with a singleton pregnancy a month is feasible. With this study we need to screen approximately 200 women a month to reach our sample size. Screening 200 women a month will result in 10.000 screened women in 4 years. Taking the 11.5^{th} percentile of short cervical length (35mm or less) will result in 1150 potential eligible women for our study. To reach our final sample size of 620 women we need a participation rate of 50 to 60%%.

Multiple pregnancies: With our previous experience from the AMPHIA and the ProTwin trial, we expect to screen 3.600 women in 36 months. Taking the 25th percentile of cervical length (38mm) will result in 900 eligible women for the trial. Assuming a 40% participation rate (from AMPHIA and ProTwin trial we know that women with a twin pregnancy are very likely to participate in a trial) we can randomize 332 women with a twin pregnancy.

Summarizing the numbers described above this will lead to:
- 628 randomisations in low risk singletons after screening 10.000
- 332 randomisations in multiples after screening 3.600

Total: 960 randomizations after screening 13.600 women

Although these numbers are impressive, we are positive that we will be able to achieve these numbers. With the network we developed during the Triple P trial, it will be relatively easy to start this trial. Most of the ultrasound centres are still measuring cervical lengths for the Triple P trial. Other centres are interested to start measuring cervical lengths after the good results of the PECEP trial and the individual patient data meta-analysis about vaginal progesterone in singleton pregnancies. Hospitals treating multiples are intrigued by the results of the ProTwin trial and are convinced that more research is needed regarding the best treatment options for multiples. The logistic network used for the AMPHIA and ProTwin trial can be used again to start this trial in multiples. Measuring cervical length around 20 weeks is part of the local protocol in the participating hospitals, which makes execution of this study also in this group of women feasible.

3. TREATMENT OF SUBJECTS

3.1 Investigational product/treatment

Arabin pessary- produced by Dr. Arabin & Co. GmbH
The pessary is made of silicone, which is soft and flexible. It is folded and put around the cervix by a simple vaginal examination without causing any pain. Different sizes are available. Per patient, the appropriate size has to be estimated by gynaecological exam. As described earlier a questionnaire evaluation of Arabin et al. found a generally positive opinion of the pessary treatment (*Arabin et al., 2003*). 75% of that group would use the pessary again and would even recommend it to others. They reported that there might be an increase of vaginal discharge. If there is an increase in discharge, women will be evaluated for vaginal infection and treated if necessary. Less frequent vaginal pain and blood loss have been reported. In case of these complains women are strongly advised to seek medical care. The gynaecologist will examine the patient and replace or remove the pessary if necessary. Previous studies using the pessary have shown no foetal adverse effects.

Although the exact mechanism of action of the cervical pessary is not known, it has been hypothesized that the pessary encompasses the cervix and changes the inclination of the cervical canal. By relieving direct pressure on the internal cervical ostium, it distributes the weight of the pregnant uterus onto the vaginal floor, retrosymphyseal osteomuscular structures and Pouch of Douglas. (*Vitsky et al. 1961*) Hence, it may prevent premature dilatation of the cervix and premature rupture of the membranes. Another possible explanation is that due to the encompassed cervix, the cervical canal is compressed and this might prevent deterioration or loss of the cervical mucus plug (CMP). During pregnancy, the cervix normally stays tightly closed with a CMP sealing the opening. The role of the cervical mucus plug as an immunological gatekeeper, protecting the foetoplacental unit against infection from the vagina, may potentially play an important role in preventing ascending infections leading to preterm delivery. (Becher 2009, Hein 2001) Our group and the participating hospitals already are experienced users of the pessary thanks to the ProTwin trial.

Utrogestan 200mg – produced by Besins Healthcare.
The study participant will be treated with Utrogestan 200mg per day, vaginal capsules for the time left till 36 weeks of gestation. Prescription of vaginal progesteron has become standard practice, since its effect has been proven for the indication of a short cervix. It has become part of the NVOG-guideline 'Preventie recidief spontane vroeggeboorte', in the module 'Progesteron voor het voorkomen van vroeggeboorte bij vrouwen zwanger van een eenling'. Utrogestan will be prescribed by the patientes gynaecologist and the prescriptions will be filled by her own pharmacy without specific administrative efforts. Daily capsules will be self-administered vaginally from time of randomisation up to 36 weeks gestational age or delivery, whatever comes first. Our group also has experience with vaginal progesterone through the Triple P trial.

4. INVESTIGATIONAL PRODUCT

4.1 Name and description of investigational product(s)

A. Arabin vaginal pessary, produced by Dr. Arabin & Co.
B. Utrogestan, produced by Besins Healthcare

4.2 Summary of findings from non-clinical studies

Not applicable for Arabin pessary. We refer to the Summary of Product Characteristics.

4.3 Summary of findings from clinical studies

In a study by Fonseca et al. 2007 (NEJM), vaginal progesterone was used in healthy singleton pregnancies with a short cervical length defined as less than 15 mm. Spontaneous delivery before 34 weeks of gestation was less frequent in the progesterone group than in the placebo group (19.2% vs. 34.4%;relative risk, 0.56; 95% confidence interval (CI), 0.36 to 0.86). Progesterone was associated with a non-significant reduction in neonatal morbidity (8.1% vs. 13.8%; relative risk, 0.59; 95% CI, 0.26 to 1.25; P = 0.17). There were no serious adverse events associated with the use of progesterone. The authors concluded that in women with a short cervix, treatment with progesterone reduces the rate of spontaneous early preterm delivery. However, as a significant effect on neonatal outcome was lacking, additional data are needed.

The effectiveness of progesterone agents was also addressed in a recent meta-analysis by *Sanchez-Ramos et al*. Relative to women allocated to receive placebo, those who received progestational agents had lower rates of preterm delivery (26.2% versus 35.9%; OR 0.45, 95% CI 0.25-0.80).

The number needed to treat to prevent one premature delivery was 10 (95% CI 6-24). Relative to women allocated to receive placebo, those who received progestational agents (17(alpha)-hydroxyprogesterone caproate and other forms of progesterone) also had lower rates of perinatal mortality (14.8% versus 17.1%; OR 0.69, 95% CI 0.38-1.26). Identical results were obtained for 17(alpha)-hydroxyprogesterone caproate (3.8% versus 5.2%; OR 0.69, 95% CI 0.38-1.3).

Romero et al. published in January 2012 an individual participant data meta-analysis of all trials available at that time concerning singletons with a short cervical length and progesterone. Five trials (*Cetingoz et al 2011, Fonseca et al 2007, Hassan et al 2011, O'Brien et al 2007, and Rode et al 2011*) of high quality were included with a total of 775 women and 827 infants. Treatment with vaginal progesterone was associated with a significant reduction in the rate of preterm birth <33 weeks (relative risk (RR), 0.58; 95% confidence interval (CI), 0.42– 0.80), <35 weeks (RR, 0.69; 95% CI, 0.55– 0.88), and <28 weeks (RR, 0.50; 95% CI, 0.30–0.81) composite neonatal morbidity and mortality (RR, 0.57; 95% CI, 0.40–0.81).

The Cochrane review from 2009 showed for women with a history of preterm delivery a favourable effect of progesterone administration (*Su et al., 2010*). Four studies were included involving a total of 1329 women with a past history of spontaneous preterm birth (*da Fonseca 2003; Johnson 1975; Meis 2003; O'Brien 2007*), of which two compared weekly intramuscular injection with placebo (Johnson 1975; Meis 2003), and two compared nightly vaginal progesterone with placebo (da Fonseca 2003; O'Brien 2007).

Progesterone was associated with a statistically significant reduction in the risk of preterm birth less than 34 weeks' gestation (one study; 142 women; risk ratio (RR) 0.15; 95% confidence interval (CI) 0.04 to 0.64); preterm birth less than 37 weeks' gestation (four studies; 1255 women; RR 0.80; 95% CI 0.70 to 0.92); infant birth weight less than 2500 grams (two studies; 501 infants; RR 0.64; 95% CI 0.49 to 0.83). An individual patient data (7536 children) meta-analysis of our group on thirteen trials in multiple pregnancies using progestagens (intramuscular 17-hydroxyprogesterone caproate (17Pc) or vaginal progesterone) shows no overall effect of progestagens on adverse perinatal outcome in twin pregnancies (RR 1.1; 95% CI 0.97-1.4 for 17Pc and RR 0.97; 95% CI 0.77-1.2 for vaginal progesterone) (*Schuit et al., 2012*). However, vaginal progesterone showed to reduce adverse perinatal outcome among women with a cervical length equal or below 25mm measured at randomisation or before 24 weeks of gestation (15/56 vs. 22/60; RR 0.57; 95% CI 0.47-0.70 and 14/52 vs. 21/56; RR 0.56; 95% CI 0.42-0.75, respectively).

The PECEP trial (*Goya et al., 2012*) showed that cervical pessary, in women with a singleton pregnancy and short cervical length (<25 mm), reduced preterm birth before 34 weeks (6% versus 27%). Within the Dutch obstetric research consortium, we finished the ProTwin trial (projectnr:50-50110-96-618) which studied the effectiveness of pessary in unselected twin pregnancies. Although there was no overall treatment effect of the pessary, the pessary significantly reduced preterm delivery rates in a pre-specified subgroup of women with a cervix less than the 25th percentile (38 mm) (11% vs. 25%, RR 0.44; 95%CI 0.20-0.98 for delivery less than 32 weeks). More importantly, adverse perinatal outcome rate was substantially lower in the pessary group as compared to no intervention (7% vs. 30%, RR 0.23 95% CI; 0.09-0.60).

4.4 Summary of known and potential risks and benefits

In a large individual patient data analysis on thirteen trials, in the subgroup op vaginal progesterone no difference was observed in adverse neonatal outcome between exposed and non-exposed infants. (Schuit et. al, 2014). As described earlier in section 3.1 previous studies showed no fetal adverse effects of a pessary.

4.5 Description and justification of route of administration and dosage

Micronized progesterone 200 mg can be administered either orally or vaginally, but the latter route is preferable because of enhanced bioavailability and the absence of undesirable side effects, such as sleepiness, fatigue, and headaches (*Miles et al. 1994, Friedler et al. 1999*).
Arabin cervical pessary is placed vaginally.

4.6 Dosages, dosage modifications and method of administration

See section 3.1 and 4.1

4.7 Preparation and labelling of Investigational Medicinal Product

Utrogestan, produced by Besins Healthcare.
Utrogestan is produced by original manufacturer and is authorized in the concerned Member State. No labelling is done as Utrogestan is licensed and will be filled by patients own pharmacy. Utrogestan will be reimbursed by the basic healthcare insurance. As the basic healthcare does not cover Utrogestan it can reimburse at the sponsor.

Utrogestan will be prescribed by the patients gynaecologist and the prescriptions will be filled by her own pharmacy without specific administrative efforts. Daily capsules will be self-administered vaginally from time of randomisation up to 36 weeks gestational age or delivery, whatever comes first.

Arabin vaginal pessary, produced by Dr. Arabin & Co.
The three different sizes pessaries will be labelled 'Quadruple P study".

4.8 Drug accountability

Utrogestan:

Utrogestan will be prescribed by the patients gynaecologist and the prescriptions will be filled by her own pharmacy without specific administrative efforts. The start and stop date will be documented in the drugs accountability form, as well as the batch number and expiry date. The delivery date(s), start and stop date will be collected in the CRF. This way compliance will be monitored. The study participants will also be asked to keep up their daily use in a medication dairy. The study participant will be asked to hand in this medication diary after delivery or 36 weeks of gestation, whatever comes first.

Pessary:
The pessary will be placed directly after randomization. An accountability log will be filled in to register the size, LOT number, expiration date and date of placement and removement.

5. METHODS

5.1 Study parameters/endpoints

5.1.1 Main study parameter/endpoint

Primary outcome is composite adverse perinatal outcome. This composite outcome contains periventricular leukomalacia (PVL) > grade 1, severe respiratory distress syndrome (RDS) bronchopulmonary Dysplasia (BPD), Intraventricular Haemorrhage (IVH) grade III or IV, Necrotizing Enterocolitis (NEC), proven sepsis, (intrapartum) stillbirth and death before discharge from the nursery.

5.1.2 Secondary study parameters/endpoints (if applicable)

Secondary outcome measures are time to delivery, preterm birth rate before 28, 32, 34 and 37 weeks, (days of) admission in neonatal intensive care unit, maternal morbidity specifically thrombo-embolic

complications, infections, pneumonia, endometritis, eclampsia/HELLP, maternal death and admission days for preterm labour, and costs.
Other study parameters: The outcomes of patients outside the trial will be monitored through linkage tot the national perinatal registry as described in section 2.1

5.1.3 Long term follow-up

If a sufficient budget is awarded, the possibilities to perform a long-term follow-up will be assessed. This will be added to the trial documents using an amendment. Permission to approach patients for follow-up research will be asked via the informed consent.

5.2 Randomisation, blinding and treatment allocation

GCP trained nurses will counsel patients, ask informed consent, perform randomization and collect data. Randomization will be centrally controlled using an on-line computerised randomisation service, once patient data have been entered in a web-based database. Centres will be able to access the randomisation service 24hr/day. Subjects will be randomized in a 1:1 ratio to progesterone and pessary. Randomization will be stratified by centre (to prevent any imbalance between groups in aspects of maternal or neonatal care that may differ between centres) and by singleton / multiple pregnancy. Due to the type of interventions, this study will not be blinded.

5.3 Study procedures

INTERVENTIONS: Eligible women will be randomly allocated to receive either a cervical pessary or vaginal progesterone. The cervical pessary will be placed in situ at 18 to 22 weeks in singleton pregnancies and at 16 to 22 weeks in multiple pregnancies, and will stay in situ up to 36 weeks gestation or until delivery, whatever comes first. Vaginal progesterone will be taken from time of randomisation onwards up to 36 weeks gestational age or delivery, whatever comes first. The capsules will be self-administered vaginally by patients on a daily basis.
Before entry into the study, the investigator or an authorized member of the investigational staff must explain to potential subjects the aims, methods, reasonably anticipated benefits, and potential hazards of the study. Subjects will be informed that their participation is voluntary and that they may withdraw consent to participate at any time. They will be informed that choosing not to participate will not affect the care the subject will receive. The research nurse who will obtain the informed consent can give more information.
Each subject must give written consent. The subject will be given sufficient time to read the patient information and the informed consent form and gets the opportunity to ask questions. An independent physician will be accessible for any question the subjects may have. The consent form must be signed before any study-related activity can take place. A copy of the informed consent form must be given to the subject. Women who do not want to participate in the treatment part of the study are asked to give informed consent for the observational cohort in the context of healthcare evaluation.
In all women, we will measure cervical length prior to randomisation following the procedure described under 2.1. All women participating will be enrolled after transvaginal measurement for cervical length

between a gestational age of 16 to 22 weeks. Apart from this research intervention, women are treated according to the local protocol in the participating clinics and other interventions i.e. tocolysis and corticosteroids in case of a threatened preterm birth can be carried out as usual. Furthermore, no extra interventions will be needed.

See section 3.1 and 4.1 for dosage and distribution off the study medication and pessary.

5.4 Withdrawal of individual subjects

Subjects may withdraw at any time or be withdrawn by the investigator if the woman violates the study plan or for administrative and /or safety reasons. Patients who withdraw from the study will remain in their treatment group for the final analysis according to the intention-to-treat principle. Every effort will be made to obtain complete information on each patient randomized. The only reason for not obtaining complete information is that the patient was lost to follow up or that she withdraws consent to access her medical chart after delivery. Once a woman has been randomized, even though she stops taking the study medication or stop use the medical study device for any reason, follow-up will be continued including the planned visits, maternal and fetal surveillance. If a woman refuses to complete her follow-up visits with the research nurse (RN), the RN will confirm permission to consult her hospital chart in order to be able to complete information on the primary outcome of the study. In this case, her data will be considered in the final analysis.

5.5 Premature termination of the study

The study can be stopped prematurely based on recommendations of the Data Safety Monitoring Board (6.3 and 7.4).

6. SAFETY REPORTING

6.1 Temporary halt for reasons of subject safety

In accordance to section 10, subsection 4, of the WMO, the sponsor will suspend the study if there is sufficient ground that continuation of the study will jeopardise subject health or safety. The sponsor will notify the accredited METC without undue delay of a temporary halt including the reason for such an action. The study will be suspended pending a further positive decision by the accredited METC. The investigator will take care that all subjects are kept informed.

6.2 AEs, SAEs

6.2.1 Adverse events (AEs)

Adverse events are defined as any undesirable experience occurring to a subject during the study, whether or not considered related to (the investigational product / the experimental intervention).

Due to the fact that the population is pregnant with a notable risk of premature birth, specific SAE's are to be expected and will not be reported through Toetsing Online. They will be reported to the METC by line listing every six months.

For this protocol only the following adverse events will be collected:

- Perinatal death
- Severe neonatal morbidity
- Preterm delivery before 34 weeks
- Necrosis of the cervix as a result of the pessary
- Hospitalisation or prolongation of existing inpatients' hospitalisation of the mother due to threatened preterm birth or other pregnancy induced pathology like pregnancy induced hypertension, pre-eclampsia, hydronephrosis etc.

Adverse events of special interest regardless relationship to investigational product occurring from the first study-related procedure until 30 days after delivery. Adverse events occurring after 30 days should also be reported if considered at least possibly related to the investigational medicinal product by the investigator

The AEs must be documented in an AE form and the outcome must be followed-up until the return to normal or consolidation of the patient's condition.

6.2.2 Serious adverse events (SAEs)

A serious adverse event is any untoward medical occurrence or effect that at any dose:

- Results in maternal death;
- Is life threatening (at the time of the event) to the mother;
- Requires hospitalisation or prolongation of existing inpatients' hospitalisation other than named above in the AE listing.;
- Results in persistent or significant disability or incapacity of the mother;
- Is a congenital anomaly or birth defect of the neonate; or
- Any other important medical event that may not result in death, be life threatening, or require hospitalization, may be considered a serious adverse experience when, based upon appropriate medical judgement, the event may jeopardize the subject or may require an intervention to prevent one of the outcomes listed above.

.

The following events are not considered to be a Serious Adverse Event:

- Admissions due to premature labour or threatened premature labour. Premature delivery before 34 weeks has to be reported as AE.
- Admissons due to labour or scheduled delivery/ caesarean section > 34 weeks.
- Hospitalization for a procedure that was planned prior to study participation (i.e. prior to registration or randomization). This should be recorded in the source documents. Prolonged

hospitalization for a complication of such procedures remains a reportable serious adverse event.
- Prolonged hospitalization for technical, practical, or social reasons, in absence of an adverse event.

Serious Adverse Events (SAEs) will be reported from the first study-related procedure until 30 days after delivery. Serious Adverse events occurring after 30 days should also be reported if considered at least possibly related to the investigational medicinal product by the investigator.

The expedited reporting will occur not later than 15 days after the sponsor has first knowledge of the serious adverse reactions. SAEs that result in death or are life threatening should be reported expedited. The expedited reporting will occur not later than 7 days after the responsible investigator has first knowledge of the adverse reaction. This is for a preliminary report with another 8 days for completion of the report.

6.2.3 Follow up of (serious) adverse events

All (serious) adverse events will be followed clinically until they are resolved or until a stable situation has been reached. Depending on the event, follow up may require additional tests or medical procedures as indicated, and/or referral to the general physician or a medical specialist. Follow up information on SAEs should be reported monthly until recovery or until a stable situation has been reached. The final outcome of the SAE should be reported on a final SAE report form provided.

Subjects withdrawn from the study due to one of the AEs will be followed at least until the outcome is determined even if it implies that the follow-up continues after the patient has left the trial.

6.3 Data Safety Monitoring Board (DSMB)

A DSMB will be established prior to start of the trial. The members of the DSMB are Dr J.J. Duvekot (gyaecologist-perinatologist at the Erasmus MC, (chair)), Professor K.C.B. Roes (professor in Clinical Trial Methodology at the Julius Center of the UMC Utrecht), Dr H.I.J. Wildschut (gynaecologist-perinatologist until retirement in 2016), Professor H.W. Bruinse (professor in Clinical Obstetrics at the UMC Utrecht until he retired in 2010) and dr. H. van der Lee (Clinical epidemiology, AMC, Amsterdam(secretary)).

The advice(s) of the DSMB will only be sent to the sponsor of the study. Should the sponsor decide not to fully implement the advice of the DSMB, the sponsor will send the advice to the reviewing METC, including a note to substantiate why (part of) the advice of the DSMB will not be followed.

7. STATISTICAL ANALYSIS

7.1 Primary study parameter(s)

Data will initially be analysed according the intention to treat principle. The main outcome variable will be assessed by calculating rates in the two groups, and the accompanying relative risks and 95% confidence intervals using a random intercept fixed effects binomial model with a log link function. The random intercept is used to account for the stratified randomization. Numbers needed to treat will be calculated when appropriate. To evaluate the potential of each of the strategies, we will also perform a per protocol analysis, taking into account only those cases that were treated according to protocol.
All analyses will be performed for singleton and multiple pregnancies separately. In multiple pregnancies the clustering of children within one mother will be taken into account in the analysis using generalised estimating equations instead of a log-binomial model [ref: Gates & Brocklehurst, BJOG 2004].

7.2 Secondary study parameter(s)

Time to delivery will be evaluated by Kaplan-Meier estimates, with account for different durations of gestation at entry, and will be tested with the log rank test. Again, stratified randomisation will be taken into account by incorporating centre as a stratification variable. The other secondary outcome measures will be approached similarly to the primary outcome measure. Differences in continuous outcomes between both strategies will also be assessed using a random intercept fixed effects linear regression model. We plan a pre-specified subgroup analysis based on cervical length, specifically a cervical length below 25 mm and equal to and above 25 mm, below the 25th percentile, between the 25th and 50th percentile, and above the 50th percentile. Subgroup analysis will be performed by including an interaction term between cervical length and the treatment allocation (pessary vs. vaginal progesterone). When the interaction will be found statistically significant ($p<0.05$) we will estimate the treatment effect within the different strata of the subgroup. Apart from cervical length, we will perform subgroups analyses based on parity, previous term or preterm birth, chorionicity and number of multiples.

7.3 Other study parameters

Long Term Follow Up:

If sufficient budget becomes available the possibilities to perform a long-term follow-up study will be assessed and planned. This will be added to the trial documents using an amendment. Permission to approach patients for follow-up research will be asked via the informed consent.

Cost-effectiveness analysis

For medical costs, the process of care is divided into three cost stages (antenatal stage, delivery/childbirth, postnatal stage). Health care utilization in the antenatal stage consists of admission due to threatened preterm birth, transport of patients to perinatal centres and maternal monitoring with various tests and hospital care. Costs generated during delivery are dominated by the course of childbirth and type of delivery. Resource utilization in the postnatal stage consists of maternal and

neonatal health care during hospital admission (maternal ward, medium high and intensive care) and primary care following discharge. If neonatal health at discharge is suboptimal, further direct medical, direct non-medical and indirect costs may occur. Volumes of health care resource used are measured prospectively alongside the clinical study in centers as a part of the CRF. For the costs of screening we will focus on capital and operating expenses. Capital expenses include the costs of equipment. Operating expenses include the costs for disposables and personnel. For each stage and each cost category costs are measured as the volumes of resources used multiplied with appropriate valuations (unit-costs). Unit costs will be estimated according to the Dutch guideline on (unit) costing in health care (*Hakkaart et al., 2010*). Both costs and outcomes will be discounted with 5%."

7.4 Interim analysis (if applicable)

An interim analysis for effectiveness will not be performed. In case of a strong positive effect of one of the investigated interventions, the trial will be continued. Negative effects will be detected by the data safety monitoring committee based on the SAE's in both treatment arms.
For this trial we plan an interim safety review when on the primary outcome is available for a third of the inclusions, 320 patients.

This review will focus on the following safety outcomes:

- Neonatal mortality
- Neonatal safety
    - Early preterm birth (< 32 weeks gestational age)
    - Duration of hospital stay for infant (stratified by pre-term vs. aterm)
    - Sepsis (early or late) or meningitis
- Maternal mortality
- Maternal safety
    - hospitalization not related to not related to delivery or threatened preterm birth
    - Damage to the cervix (cervical rupture or necrosis of the cervix)
- Pregnancy complications
    - PPROM <36 weeks gestational age
    - treated urinary or genital tract infections
    - Chorioamnionitis

These outcomes will be reported separately for singleton and twin pregnancies.

Serious events that may cause concern about the safety of the study (such as maternal mortality), will be reported to the DSMB immediately if they occur. On the basis of these results, the DSMB can decide to perform additional safety reviews (for example, at 2/3 of the inclusions)

ETHICAL CONSIDERATIONS

7.5 Regulation statement

The study will be conducted according to the principles of the Declaration of Helsinki (WORLD MEDICAL ASSOCIATION DECLARATION OF HELSINKI) Ethical Principles for Medical Research Involving Human Subjects Version Edinburgh, Scotland, October 2000, with Note of Clarification on Paragraph 29 added by the WMA General Assembly, Washington 2002 end Note of Clarification on Paragraph 30 added by the WMA General Assembly, Tokyo 2004 and in accordance with the Medical Research Involving Human Subjects Act (WMO) and other guidelines, regulations and Acts.

7.6 Recruitment and consent

All women with a short cervical length (singleton or multiple pregnancy) will be informed in brief about the clinical trial by the supervising gynaecologist or by the attending resident. Subsequently, a trained research nurse will inform the patient in detail. The patient will also obtain written information about the study from the research nurse. Then the patient can think about participation in the clinical trial and discuss the study with her family. In case of participation, the informed consent form should be signed prior to randomisation.

7.7 Compensation for injury

The sponsor/investigator has a liability insurance, which is in accordance with article 7, subsection 6 of the WMO.

The sponsor (also) has an insurance which is in accordance with the legal requirements in the Netherlands (Article 7 WMO and the Measure regarding Compulsory Insurance for Clinical Research in Humans of 23th June 2003). This insurance provides cover for damage to research subjects through injury or death caused by the study.

1. € 450.000,- (i.e. four hundred and fifty thousand Euro) for death or injury for each subject who participates in the Research;
2. € 3.500.000,- (i.e. three million five hundred thousand Euro) for death or injury for all subjects who participate in the Research;
3. € 5.000.000,- (i.e. five million Euro) for the total damage incurred by the organisation for all damage disclosed by scientific research for the Sponsor as ‘verrichter’ in the meaning of said Act in each year of insurance coverage.

The insurance applies to the damage that becomes apparent during the study or within 4 years after the end of the study.

8. ADMINISTRATIVE ASPECTS, MONITORING AND PUBLICATION

8.1 Handling and storage of data and documents

Data will be collected using Oracle Clinical Remote Data Capture (RDC), which is a generation of application system that enables collection and clean-up of clinical trial data using the Internet. For detailed information on Oracle RDC, please visit the page of Oracle RDC products. (http://www.ctc-

g.co.jp/~CTCLS/opa/en/). The expertise for this technology is already available in the study group and is extensively used with the Dutch Obstetrics Consortium. Data monitoring will be done by research nurses in each of the participating centres. Data handling will be done anonymously.

8.2 Amendments

Amendments are changes made to the research after a favourable opinion by the accredited METC has been given. All amendments will be notified to the METC that gave a favourable opinion.
All substantial amendments will be notified to the METC and to the competent authority.
Non-substantial amendments will not be notified to the accredited METC and the competent authority, but will be recorded and filed by the sponsor.

8.3 Annual progress report

The sponsor/investigator will submit a summary of the progress of the trial to the accredited METC once a year. Information will be provided on the date of inclusion of the first subject, numbers of subjects included and numbers of subjects that have completed the trial, serious adverse events/ serious adverse reactions, other problems, and amendments.

8.4 End of study report

The investigator will notify the accredited METC of the end of the study within a period of 8 weeks. The end of the study is defined as the last patient's last visit. In case the study is ended prematurely, the investigator will notify the accredited METC within 15 days, including the reasons for the premature termination. Within one year after the end of the study, the investigator/sponsor will submit a final study report with the results of the study, including any publications/abstracts of the study, to the accredited METC.

8.5 Public disclosure and publication policy

Results will be published in international and national journals. In the Netherlands the Committee on Quality Care of the NVOG will incorporate the results in the guideline on management of twin and multiple pregnancies. This study will be performed in at least the eight centres mentioned in this application, and at least four teaching hospitals affiliated to the eight tertiary care centres have promised to participate in the study as well.

9. STRUCTURED RISK ANALYSIS

9.1 Potential issues of concern

a. Level of knowledge about mechanism of action

Progesterone has a role in maintaining pregnancy and is thought to act by suppressing smooth muscle activity in the uterus. In many animal species, there is a reduction in the amount of circulating progesterone before the onset of labour. While these changes have not been shown to occur in women, it has been suggested that there is a 'functional' withdrawal of progesterone related to changes in the expression of progesterone receptors in the uterus.

Vaginal pessaries are used to prevent preterm birth since 1959 (Vitsky). During the pregnancy the cervix normally stays tightly closed with a cervical mucus plug (CMP) sealing the opening. It is hypothesized that impairment of the CMP for example by cervical effacement can lead to an ascending infection and preterm delivery; nevertheless, this remains to be further clarified. The vaginal pessary encompasses the cervix and compresses the cervical canal preventing deterioration of the CMP. The pessary alternates the inclination of the cervical canal and corrects the incompetent cervix pointing forward in the axis of the vagina. It relieves direct pressure on the internal cervical os by distributing the weight of the pregnant uterus onto the vaginal floor, retrosymphyseal osteomuscular structures and Douglas cavity and may so prevent premature dilatation of the cervix and premature rupture of the membranes. Furthermore, it blocks the fetal head to descend and press on the internal ostium.

b. Previous exposure of human beings with the test product(s) and/or products with a similar biological mechanism

Romero et al. published in January 2012 an individual participant data meta-analysis of all trials available at that time concerning singletons with a short cervical length and progesterone. Five trials (Cetingoz et al 2011, Fonseca et al 2007, Hassan et al 2011, O'brien et al 2007, and Rode et al 2011) of high quality were included with a total of 775 women and 827 infants. Treatment with vaginal progesterone was associated with a significant reduction in the rate of preterm birth <33 weeks (relative risk [RR], 0.58; 95% confidence interval (CI), 0.42– 0.80), <35 weeks (RR, 0.69; 95% CI, 0.55– 0.88), and <28 weeks (RR, 0.50; 95% CI, 0.30–0.81) composite neonatal morbidity and mortality (RR, 0.57; 95% CI, 0.40–0.81).

The Cochrane review from 2009 showed for women with a history of preterm delivery a favourable effect of progesterone administration. Four studies were included involving a total of 1329 women with a past history of spontaneous preterm birth (da Fonseca 2003; Johnson 1975; Meis 2003; O'Brien 2007), of which two compared weekly intramuscular injection with placebo (Johnson 1975; Meis 2003), and two compared nightly vaginal progesterone with placebo (da Fonseca 2003; O'Brien 2007).Progesterone was associated with a statistically significant reduction in the risk of preterm birth less than 34 weeks' gestation (one study; 142 women; risk ratio (RR) 0.15; 95% confidence interval (CI) 0.04 to 0.64); preterm birth less than 37 weeks' gestation (four studies; 1255 women; RR 0.80; 95% CI 0.70 to 0.92); infant birth weight less than 2500 grams (two studies; 501 infants; RR 0.64; 95% CI 0.49 to 0.83).

Newcomer performed a MEDLINE search from 1966-2000 (Newcomer J 2000). This review indicated that based on non-randomized studies a pessary is potentially useful in the prevention of premature birth and seems to be without any significant risks or side effects. The only RCT of pessary prophylaxis compared a pessary to cerclage. The type of pessary used, the entry criteria and the method of randomisation, were not well described (Förster et al. 1986). The trial recruited 112 patients in the cerclage group and 130 in the pessary group. Mean gestational age at initiation of the therapy was 27 weeks. Both methods were equal in their effects, but in view of the poor quality of the trial and the late gestational age at start of treatment, these results have little significance.
A prospective cohort study by Acharya et al. also used a double ring-shaped pessary (Arabin-pessary) to treat cervical incompetence in women with a cervical length ≤ 25mm, before 30 weeks (Acharya et al., 2006). The study showed that in 55 percent of the patients, the pregnancy could be postponed to 34 weeks or more. All other reports have been of uncontrolled case series or non-randomized comparative studies.
A double ring-shaped pessary is at present the most popular pessary. It has been evaluated in one cohort study (Arabin et al., 2004). This study showed in women with a twin pregnancy a mean gestational age at delivery 35+6 (33-37+4) in the pessary group (n=23) and 33+2 (24+4-37+2) in control group (n-23) (p=0.02). From the 23 women with a pessary, no one delivered before 32 weeks versus 8 women in the control group (P < .001, RR 0.12, 95% CI 0.02 to .88). Based on this study, the Arabin pessary is widely used in The Netherlands and Germany with the manufacturers selling around 2000 per year.
In april 2012 the results of the PECEP trial were published (Goya et al.). This RCT included women with singleton pregnancies who have at ultrasound examination at 18+0 to 22+6 weeks of gestation a short cervix. Patients were randomized to a pessary or no intervention. The proportion of spontaneous deliveries before 34 weeks, the trial's primary endpoint, was reduced in women who received a cervical pessary (6% vs. 27%; odds ratio 0·18, 95% CI 0·08–0·37). These results are very impressive and promising.

c. Can the primary or secondary mechanism be induced in animals and/or in *ex-vivo* human cell material?

In many animal species, there is a reduction in the amount of circulating progesterone before the onset of labour.

d. Analysis of potential effect

Whilst no teratogenic effects have been described with most progesterone, there is little in the way of long-term safety data. Maternal side-effects from progesterone therapy include headache, breast tenderness, nausea, cough and local irritation if administered intramuscularly. At present, there is little information available regarding the optimal dose of progesterone, mode of administration, gestation to commence therapy, or duration of therapy (Iams 2003).

e. Pharmacokinetic considerations

Resorption: 60% through intestinal pathway as progesterone and -metabolites. F = 6–10% by high first-pass-effect. T_{max} = 1–4 hours. Most important metabolites: active 20-α-dihydroxyprogesteron and inactive pregnandiol. Elimination: mostly with urine.

f. Study population

Women with a singleton or twin pregnancy undergoing foetal assessment at 16 to 22 weeks will be offered cervical length measurement. Women with a short cervix (singleton pregnancy 35 mm or less (11,5nd percentile), twin less then 38 mm (25th percentile), will be invited to participate in a randomized clinical trial.

g. Can effects be managed?

Treatment with the cervical pessary or vaginal progesterone can both be stopped. The effect of the pessary will disappear directly. There is no antidote for progesterone, due to the high first-pass-effect we believe that the effect will be gone relatively fast.

9.2 Synthesis

The 17-alpha-hydroxyprogesterone (17-OHPC) caproate is a natural progesterone that is produced by the placenta. Although masculinisation of the genital tract in female foetuses was feared in the past no such side effect has been found after thorough follow-up even when 17-OHPC was administered in early pregnancy. The same applies for the incidence of hypospadics in male infants. No difference was observed between exposed and non-exposed infants (*Resseguie et al., Yovich et al., Katz et al.*) Arabin et al performed comparable research, showing in a questionnaire evaluation within the treatment group a general positive opinion of the treatment (*Arabin et al., 2003*). 75 % of that treatment group would use the pessary again and would even recommend it to others. They reported that there might be some increase of vaginal discharge. If there is an increase in discharge, women will be evaluated for vaginal infection and treated if necessary. Previous studies using the pessary have shown no foetal adverse effects.

So neither the cervical pessary and progesterone are associated with increased neonatal or maternal morbidity and mortality. In our opinion women participating in this study are not at increased risk.

10. REFERENCES

Arabin B, Halbesma JR, Vork F, Hubener M, van EJ. Is treatment with vaginal pessaries an option in patients with a sonographically detected short cervix? J Perinat Med 2003;31:122-33

Acharya G, Eschler B, Gronberg M, Hentemann M, Ottersen T, Maltau JM. Noninvasive cerclage for the management of cervical incompetence: a prospective study. Arch Gynecol Obstet 2006 Feb;273(5):283 ‐ 7.

Becher N, Adams WK, Hein M, Uldbjerg N. The cervical mucus plug: structured review of the literature. Acta Obstet Gynecol Scand 2009;88(5):502-513.

Clements KM, Barfield WD, Ayadi MF, Wilber N. Preterm birth-associated cost of early intervention services: an analysis by gestational age. Pediatrics 2007;119:e866-74.

da Fonseca EB, Bittar RE, Carvalho MH, Zugaib M. Prophylactic administration of progesterone by vaginal suppository to reduce the incidence of spontaneous preterm birth in women at increased risk: a randomized placebo-controlled double-blind study. Am J Obstet Gynecol 2003;188:419-24

Fowlie PW and McHaffie H. Supporting parents in the neonatal unit. BMJ 2004;329:1336-38

Goya M, Pratcorona L, Merced C, Rodó C, Valle L, Romero A, Juan M, Rodríguez A, Muñoz B, Santacruz B, Bello-Muñoz JC, Llurba E, Higueras T, Cabero L, Carreras E; on behalf of the Pesario Cervical para Evitar Prematuridad (PECEP) Trial Group. Cervical pessary in pregnant women with a short cervix (PECEP): an open-label randomized controlled trial. Lancet 2012 epub ahead of print

Hegeman MA, Bekedam DJ, Bloemenkamp KW, Kwee A, Papatsonis DN, van der Post JA, Lim AC, Scheepers HC, Willekes C, Duvekot JJ, Spaanderman M, Porath M, van Eyck J, Haak MC, van Pampus MG, Bruinse HW, Mol BW. Pessaries in multiple pregnancy as a prevention of preterm birth: the ProTwin Trial. BMC Pregnancy Childbirth 2009;9:44

Hein M, Helmig RB, Schonheyder HC, Ganz T, Uldbjerg N. An in vitro study of antibacterial properties of the cervical mucus plug in pregnancy. Am J Obstet Gynecol 2001;185(3):586-592.

Iams JD, Goldenberg RL, Meis PJ, et al. The length of the cervix and the risk of spontaneous premature delivery. N Engl J Med 1996;334:567-72

Jodie M Dodd, Vicki Flenady, Robert Cincotta, Caroline A Crowther. Prenatal administration of progesterone for preventing preterm birth in women considered to be at risk of preterm birth Cochrane Database Syst Rev. 2009 apr 15

Liem SMS, Schuit E, Hegeman MA, Bais AG, de Boer K, Bloemenkamp KWM et al. Cervical pessaries for prevention of preterm birth in woman with a multiple pregnancy (ProTWIN); a multicentre, open-label randomised controlled trial. The Lancet 382; 9901;1341-1349.

Lim AC, Bloemenkamp KW, Boer K, Duvekot JJ, Erwich JJ, Hasaart TH, et al. Progesterone for the prevention of preterm birth in women with multiple pregnancies: the AMPHIA trial. BMC Pregnancy Childbirth 2007;7:7

Litjens JTM, Oude Rengerink K, Danhof NA, Kruitwagen RFPM, Mol BW Does recruitment for clinical trials improve dissemination and timely implementation of their results – A questionnaire survey Clinical Trials Conditionally accepted for publication

Mol BW, Ruifrok AE; for the Global Obstetrics Network. Global Alignment, Coordination and Collaboration in Perinatal Research: The Global Obstetrics Network (GONet) Initiative. Am J Perinatol. 2012 Aug 14.

Meis PJ, Klebanoff M, Thom E, et al: National Institute of Child Health and Human Developement Maternal-Fetal Medicine Units Network. Prevention of recurrent preterm delivery by 17 alpha-hydroxyprogesterone caproate. N Engl J Med 2003;348:2379-85

Newcomer J. Pessaries for the treatment of incompetent cervix and premature delivery. Obstet Gynecol Surv 2000 Jul;55(7):443 ‐ 8.

van Os MA, van der Ven JA, Kleinrouweler CE, Pajkrt E, de Miranda E, van Wassenaer A, Porath M, Bossuyt PM, Bloemenkamp KW, Willekes C, Woiski M, Oudijk MA, Bilardo KM, Sikkema MJ, Duvekot JJ, Veersema D, Laudy J, Kuiper P, de Groot CJ, Mol BW, Haak MC. Preventing preterm birth with progesterone: costs and effects of screening low risk women with a singleton pregnancy for short cervical length, the Triple P study. BMC Pregnancy Childbirth 2011;11:77

Romero R, Nicolaides K, Conde-Agudelo A, et al. Vaginal progesterone in women with an asymptomatic sonographic short cervix in the midtrimester decreases preterm delivery and neonatal morbidity: a systematic review and metaanalysis of individual patient data. Am J Obstet Gynecol 2012;206:124.e1-19

Sanchez-Ramos L, Kaunitz AM, Delke I. Progestational agents to prevent preterm birth: a meta-analysis of randomized controlled trials. Obst Gynecol 2005;105:273-9

Schuit E, Stock S, Groenwold RH, Maurel K, Combs CA, Garite T, et al. Progestogens to prevent preterm birth in twin pregnancies: an individual participant data meta-analysis of randomized trials. BMC Pregnancy Childbirth 2012;12:13

Schaaf JM, Mol BW, Abu-Hanna A, Ravelli AC. Trends in preterm birth: singleton and multiple pregnancies in the Netherlands, 2000-2007. BJOG. 2011 Sep;118(10):1196-204.

Su LL, Samuel M, Chong YS. Progestational agents for treating threatened or established preterm labour. Cochrane Database Syst Rev 2010;1:CD006770

Vitsky M. Simple treatment of the incompetent cervical os. Am J Obstet Gynecol 1961 Jun;81:1194-7.
