# Supplementary material for: Cervical pessary versus vaginal progesterone in women with a multiple pregnancy and a short cervix: A randomised controlled trial
Source: PLoS Med. 2025 Nov 3;22(11):e1004586. doi: 10.1371/journal.pmed.1004586 (PMC12591417; doi:10.1371/journal.pmed.1004586)
Supplement: S3 File — (PDF) [file pmed.1004586.s003.pdf]

-Quadruple P Study-

Multiples

Pessary or Progesterone to Prevent Preterm delivery in multiples without a history of spontaneous preterm birth < 34 weeks of GA and with short cervical length

Statistical Analysis Plan

EUDRA CT no.<br><br>Dutch Clinical trial registry no.	EUCTR2013-002884-24-NL<br>Date of registration: December 5th 2013<br><br>*Former Trial registration number: NTR 4414.*<br>*Date of registration January 29th 2014*
Principal investigator, centre	Prof.dr. E. Pajkrt, gynecologist
Coordinating investigator	Prof. dr. E. Pajkrt, gynecologist
Sponsor	Amsterdam University Medical Centre
Funding	Stichting Stop te vroeg bevallen
SAP version, date	Version 1, 4th of October 2023<br><br>Final version, 5th of June 2024
Trial methodologist	M.C. van der Weide
SAP author	C.E. van Dijk

Names and signatures

Please obtain dated signatures from all contributors, including the principal investigator and methodologist/statistician when a new version of the statistical analysis plan has been completed, approved by the principal investigator and other researchers and formally filed (for example in the trial master file).

Role of contributor	Name and full affiliation	Signature	Date of signature
Principal investigator	E. Pajkrt, prof, PhD, MD		08-06-2024
Researcher who will perform the statistical analysis	C.E. van Dijk, MD		05-06-2024
Methodologist/statistician consulted	M.C. van der Weide, PhD		05-06-2024
Contributor to statistical analysis plan	B.W. Mol, prof. PhD, MD<br>B.M. Kazemier, PhD, MD<br>A.L. van Gils, MD		06-06-2024<br>07-06-2024<br>06-06-2024

Revision history of statistical analysis plan

The revision history of the statistical analysis plan includes a version number, date of approval, summary of changes, justification of revision and timing of the revisions with respect to changes to the protocol, data safety monitoring board meetings, interim analyses and the final analyses. The revision history should be formally filed (for example in the trial master file). It does not need to contain all versions made in the internal process of producing a new filed version.

Updated statistical analysis plan version	Protocol version	Section number(s) changed	Description of and reason for changes	Date of approval
1.0	V1	Initial analysis plan	First draft	04-10-2023
2.0	V2	Adjustments to secondary endpoints and specification of analyses (adjusted for twins), sample size explanations and tables.	Finetuning in alignment with protocol	05-06-2024

1. Introduction

1.1 Background

Preterm birth (PTB) is in quantity and in severity the most important issue in obstetric care in the developed world. Of all perinatal mortality, 50% to 70% can be attributed to PTB, with increased mortality rates for those being delivered more prematurely. In women with a singleton pregnancy, the spontaneous (s)PTB rate in The Netherlands is just under 5%, resulting in a perinatal mortality rate of 0.8% and a severe disability rate of 0.7%,while 2% of the children suffer from moderately disability (Schaaf et al., 2011).

Spontaneous preterm delivery is the single most important cause of perinatal mortality in the western World, and prevention of PTB is a major goal of obstetrical care. Ultrasonographic measurement of cervical length at 20-23 weeks of gestation identifies women at risk for PTB (Iams et al, 1996). During pregnancy, most women do not give serious consideration to the possibility of preterm delivery. In most cases, admission of an infant to the neonatal unit is unexpected and is stressful for the parents (Fowlieet al., 2004). Until recently, preventive treatments were not available. However, in the last decade two important breakthroughs have been established. Progestogens and cervical pessaries are both considered as potential preventive treatments in women with a short midtrimester cervix.

1.2 Rationale

A short cervix measured at the second trimester identifies women at risk for preterm birth and both progestogens and cervical pessary are promising treatments for women who are at increased risk for PTB due to a short cervix. When this trial was started, no study had compared cervical pessary with progesterone in low-risk multiples with a short cervix.

1.3 Objective

A randomised controlled trial will be performed in individuals pregnant with a multiple pregnancy, no history of sPTB before 34 weeks gestation and with a short cervix of ≤37mm measured between 16-22 weeks' gestation. Subsequently, we will randomise women to cervical pessary or vaginal progesterone. The data will be used to assess if a cervical pessary is superior to vaginal progesterone in the prevention of adverse perinatal outcomes resulting from preterm delivery.

2. Endpoints

2.1 Primary endpoint

Primary outcome is a composite of adverse perinatal outcomes. For this primary outcome to be true, one or more of the following composite outcomes need to be found:

- Periventricular leukomalacia (PVL) grade II or more yes/ no
- Chronic Lung Disease* yes/ no
- Intraventricular Haemorrhage (IVH) grade III or IV yes/ no
- Necrotizing Enterocolitis (NEC grade II or more yes/ no
- Retinopathy of prematurity (ROP) yes/ no
- Proven sepsis (early or late) yes/ no
- (intrapartum) Stillbirth and death before discharge from the nursery yes/ no

⇨ All measured up until 10 weeks after the expected due date

⇨ Assessed at both maternal (indicating that the primary outcome is considered valid if either one or both of the fetuses experience an adverse outcome) and neonatal level.

* Severe respiratory distress syndrome (RDS) or Bronchopulmonary Dysplasia (BPD).

2.2 Secondary endpoints

- Time to delivery (days)
- Preterm birth rate before 24, 28, 32, 34 and 37 weeks (n/N, %)
  - (spontaneous and total)
- Birth weight (total, below 1500gr, below 2500gr) (gram, yes/no)
- Patent ductus arteriosus (PDA) (yes/no)
- Treated seizures (yes/no)
- Premature rupture of the membranes (PPROM) (yes/no)
- (days of) Admission in neonatal intensive care unit (days)
- Maternal morbidity (intra- en postpartum):
  - thromboembolic complications (yes/no)
  - eclampsia/HELLP (yes/no)
  - chorioamnionitis (yes/no)
  - urinary tract infections (yes/no)
  - pneumonia (yes/no)
  - endometritis (yes/no)

  - death (yes/no)
- Mode of delivery (vaginal/ assisted/caesarean section)
- Twin-to-twin Transfusion Syndrome (TTS) (yes/no)
- All components of the composite outcome will also be assessed separately as secondary outcome.

3. Study methods

3.1 Study design

Multicentre randomised controlled study. It is a superiority trial comparing the effectiveness of a cervical pessary compared to vaginal progesterone in the reduction of adverse perinatal outcomes.

3.2 Study population

Individuals with a multiple pregnancy between 16 and 22 weeks of gestation are offered the possibility to screen for a short cervix to identify an increased risk of preterm delivery. Their cervical lengths will be measured during the routine ultrasound check-ups from 16 weeks of gestation, after consent. If the cervical length is below the 25th percentile (≤37 mm) and there are no clinical symptoms such as vaginal blood loss or contractions etc., participation in the randomised trial is proposed. Individuals who are eligible for the study, will be asked to give informed consent prior to randomisation and participation in the study. In consultation with the treating gynaecologist, women who declined participation in the trial will be offered treatment according to local protocol.

3.3 Inclusion criteria

1. Multiple pregnancy and cervical length at 16 to 22 weeks of 37mm or less (≤37mm)
2. Written informed consent

3.4 Exclusion criteria

1. Cervical cerclage in this pregnancy
2. Maternal age less than 18 years
3. Identified major congenital abnormalities in this pregnancy*
4. Death of one or both of the foetuses in this pregnancy
5. Spontaneous preterm birth < 34 weeks in previous pregnancies
6. Participation Quadruple P study in previous pregnancies
7.Cervical length < 2mm or ≥ 38mm
8. Cervical dilatation ≥ 3cm
9. Signs of blood loss or contractions at time of randomisation

* major congenital abnormalities: defined as conditions of prenatal origin that are present at birth, potentially impacting an infant's health, development and/or survival.

3.5 Treatment of subjects

Eligible women will be randomly allocated to receive either a cervical pessary or vaginal progesterone. The cervical pessary will be placed at 16 to 22 weeks of gestation during physical examination, and will stay in situ up to 36 weeks gestation or until delivery, whatever comes first. The capsules of progesterone will be self-administered vaginally by the participants on a daily basis until 36 weeks of gestation or delivery, whatever comes first. Until 2020 distribution of progesterone was organized by the trial pharmacy and suppletion by Besins. After 2020 the vaginal progesterone was prescribed by the gynaecologist and supplied by the local pharmacy. Apart from this research intervention, participants are treated according to the local protocol in the participating clinics and other interventions i.e. tocolysis and corticosteroids in case of a threatened preterm birth can be carried out as usual.

3.6 Blinding

Due to the type of interventions, this study will not be blinded.

3.7 Randomisation procedure

Counselling and informed consent will be conducted by Good Clinical Practice trained nurses and doctors. Randomisation will be centrally controlled using an 24hr/7day accessible on-line computerized randomisation service (*ALEA Clinical software version 16 (FormsVision, Abcoude, The Netherlands) and from July 2022 in Castor Electronic Data Capture v2022.3.2.0)*. Subjects will be randomised in a 1:1 ratio to progesterone and pessary stratified by centre (to prevent any imbalance between groups in aspects of maternal or neonatal care that may differ between the in total 20 centres).

4. Sample-size

In multiple pregnancies, we expect a relative reduction of 50% of adverse perinatal outcome in the pessary group: from 24% in the vaginal progesterone group to 12% in the pessary group.

The expected percentage of adverse perinatal outcomes of the vaginal progesterone group is based on the IPD meta-analysis of Schuit et al., where they found an adverse outcome rate of 25% in a population with a short cervix of <25 mm. Since we include patients with a longer cervical length and thus less at risk, we expect slightly less adverse perinatal outcomes in the progesterone group (24% instead of 25%).

For the pessary group, we based our expected percentage on the ProTwin trial, where the adverse neonatal outcome was 12% in a comparable population with a pessary for a similar cervical length. Since twin gestations are followed up in the hospital, we expect less patients being lost to follow up. Using a two-sided test with a type I error of 5% and type II error of 20% and a loss to follow-up of 5%, we calculated we would need a sample size of 332 women (166 per group).

5. Analysis considerations

5.1 Analysis populations

5.1.1 Full Analysis Population (ITT)

Data will initially be analysed according the intention to treat principle. It includes all randomised participants who gave a signed informed consent for the study, regardless of protocol deviations or additional therapies, like a cervical cerclage. In the intention to treat analysis, participants will be analysed in groups according to the allocated treatment at randomisation. Participants who withdraw from the study will remain in their treatment group for the final analysis according to the intention-to-treat principle. Every effort will be made to obtain complete information on each participant randomised. The only reason for not obtaining complete information is that the participant was lost to follow up or that consent to access her medical chart after delivery was withdrawn. Randomised participants who appear to fail inclusion and exclusion criteria (eligibility violations) during blinded data review, will be excluded from this analysis. This will only be done for criteria that were present at the time of randomisation. Two clinicians will review such cases and where there are discrepancies, a third will be consulted. The following protocol violations will be considered:

- Missing informed consent and inadequate reporting in electronic patient file of process of counselling, giving informed consent and randomisation
- Cervical length measurements before 16+0 weeks of after 22+0 weeks of gestation
- Cervical dilatation of 3cm or more
- Cervical length measures below 2mm or above 37 mm
- Cervical cerclage before inclusion
- Symptoms of threatened preterm birth (blood loss or contractions)
- Maternal age less than 18 years
- Identified major congenital abnormalities (which are known to be associated with an increased risk for premature birth)
- Death of the foetus before inclusion/ randomisation
- Previous spontaneous preterm birth before 34 weeks of gestation
- Participation in Quadruple P study in previous pregnancy
- Fetal distress at inclusion/ randomisation

5.1.2 Per Protocol Population

To evaluate the potential of each of the strategies, we will also perform a per protocol analysis, taking into account only those cases that were treated according to protocol, which is:

- A participant meets all inclusion and exclusion criteria.

- The randomised treatment has been continued preferably up to 36 weeks of gestation or until (threatened) preterm delivery, whichever came first.
    - In case of progesterone, 100% of the possible days that medication could have been administered (PCD), is taken.
    - In case of a pessary, the device must have been in place 100% of the days it was possible to have it inserted.
    - If the treatment is continued until one day before delivery, it is also counted as 100%, as the cutoff is based on date and not on time, and a participant must also be given the time to give birth.
- No cross-over to the other treatment modality has taken place.
- No cerclage has been placed after randomisation.

5.2 Covariates and Subgroups

For the primary outcome the following subgroup analysis are pre-specified in the protocol:

- Cervical length below and equal to 25 mm (≤25 mm) compared to cervical length above 25mm
- Multiparous women without previous PTB compared to multiparous women with previous PTB and nulliparous women (multiparous women without PTB as reference).
- Monochorionicity compared to dichorionicity.
- 3 or more foetus compared to 2 foetus
- GA ≤36+0 weeks or above 36 weeks GA

For the following secondary outcomes will also be analysed for the subgroup of cervical length (≤25 mm) compared to > 25mm and for the subgroup GA ≤36+0 or above 36weeks GA:

- Spontaneous PTB rate before 34 weeks of GA (n/N, %)
- Spontaneous PTB rate before 28 weeks of GA (n/N, %)
- Time to delivery (Kaplan Meier curve)

Subgroup analysis will be performed by including an interaction term with the treatment allocation (cervical pessary vs. vaginal progesterone). When the interaction will be found statistically significant ($p<0.05$) we will estimate the treatment effect within the different strata of the subgroup.

To investigate whether a margin can be found in the intensity of treatment, we will perform a sensitivity analysis in the per protocol group by looking at 60-100% PCD.

5.3 Missing Data

When data of the delivery and follow-up of the neonate cannot be obtained, the participant will be categorized as lost to follow up and not be included in both intention to treat and the per protocol analyses. In all other

cases, the participant will be included in the intention to treat analyses and depending on the missing values in the per protocol analyses.

5.4 Interim Analyses and Data Monitoring

An interim analysis for effectiveness is not performed, only a best case scenario analysis. In case of a strong positive effect of one of the investigated interventions, the trial will be continued. Negative effects will be detected by the data safety monitoring committee based on the SAE's in both treatment arms. Serious events that may cause concern about the safety of the study (such as maternal mortality), will be reported to the DSMB immediately if they occur. A extended DSMB charter is available with all the information possible on safety analyses. There have been 4 safety interim analysis so far. The data was only observed for safety and there were no stopping rules based on the statistical significance of the effect of the treatment.

6. Efficacy analyses

6.1 Timing of final statistical analysis

The statistical analysis for the primary outcome (composite adverse perinatal outcome) and preterm birth rate before 28, 32, 34 and 37 weeks (spontaneous, iatrogenic and total) will be performed after delivery of the last child. After data cleaning for all outcomes has been completed, the primary outcome and all secondary outcomes will be analysed. It is expected that this will occur within four months of the birth of the last child.

6.2 Primary endpoint analysis

The primary outcome, the adverse perinatal outcome on child level, will be presented in prevalence rates with relative risks and 95% confidence intervals and p-values using a generalized estimating equations with log link and binomial distribution estimating both crude rates and adjusted rates with center as fixed covariate and mother as random effect to account for clustering of the multiples. A significance level of 5% will be used. There will be no adjustment of p-values, as no interim analysis for efficacy was performed.
A sensitivity analysis with a mixed model using a random effect for center will be conducted to test the robustness of the primary analysis with center as fixed covariate, thereby determining the justification for extrapolating outcomes to other centers and assessing their generalizability.

6.3 Secondary analyses

- If the difference in relative risk of the primary outcome between crude and adjusted for center is less than 10%, adjustment for center will not be applied as covariate in the secondary analyses.
- All statistical tests will use a 2-sided p-value of 0.05. All confidence intervals presented will be 95% and two-sided.

- Numbers needed to treat will be calculated when appropriate.
- To evaluate the potential of each of the strategies, a per protocol analyses will be performed, taking into account only those cases that were treated according to protocol. The per protocol selection is explained in section 5.1.2.

6.3.1 Maternal level

- Secondary outcomes concerning maternal or obstetric outcomes will be assessed for the total population of mothers, meaning the included and randomised pregnant individuals. Assessment will be done using a generalized linear log-binomial model. The composite adverse perinatal outcome will also be assessed at maternal level.
- For dichotomous outcomes, generalized linear regression analysis using a log link will be performed to calculate Relative Risks with 95% confidence intervals and corresponding p-value. When there are ≤5 events for a variable, Fisher's exact test will be used to calculate the p-value.
- The distribution of continuous outcomes will be inspected visually. For approximately normally distributed continuous outcomes, means and standard deviations will be reported and difference in means will be calculated. Mean differences and the corresponding 95% confidence intervals will be presented along with the p-value from the t-test. For highly skewed continuous outcomes, medians and interquartile ranges will be reported together with the p-value.
- Time to delivery will be evaluated by Kaplan-Meier estimates, with account for different durations of gestation at entry, and will be tested with the log rank test.

6.3.2 Neonatal level

- Neonatal outcomes will be assessed for total neonatal population (i.e. at the child level) and assessment will be done with generalized estimating equations to account for clustering within the mother.
- For dichotomous outcomes, generalized estimating equations to account for clustering within the mother will be performed to calculate Relative Risks. The 95% confidence intervals and the p-value will also be presented. When there are ≤5 events for a variable, Fisher's exact test will be used to calculate the p-value.
- The distribution of continuous outcomes will be inspected visually. For approximately normally distributed continuous outcomes, means and standard deviations will be reported and difference in means will be calculated. Mean differences and the corresponding 95% confidence intervals will be presented along with the p-value from the t-test. For highly skewed continuous outcomes, medians and interquartile ranges will be reported together with the p-value.

6.4 Other analyses

- To investigate whether a margin can be found in the intensity of treatment, we will perform a sensitivity analysis of the per protocol population by looking at 60-100% PCD.

- At maternal level, we will explore the PTB rates (spontaneous and total) of PTB before 37, 34, 32, 28 and 24 weeks of gestation within the cervical length ranges of ≤25 and above 25mm.

7. Safety analyses

All serious adverse events (SAE's) occurring during the study will be listed individually in the supplementary information. Listings will be divided according to treatment group. All serious adverse events that are considered to be possibly related to the study medication by the investigators will be marked.

7.1 Deaths, Serious Adverse Events and other Significant Adverse Events

A serious adverse event (SAE) is any untoward medical occurrence or effect that at any dose:

- Results in maternal death;
- Is life threatening (at the time of the event) to the mother;
- Requires hospitalization or prolongation of existing inpatients' hospitalization other than expected obstetric complications (such as threatened premature labour, admissions due to labour or scheduled delivery/ caesarean section);
- Results in persistent or significant disability or incapacity of the mother;
- Is a severe congenital anomaly or birth defect of the neonate; or
- Any other important medical event that may not result in death, be life threatening, or require hospitalization, may be considered a serious adverse experience when, based upon appropriate medical judgement, the event may jeopardize the subject or may require an intervention to prevent one of the outcomes listed above.

8. Comparison to study protocol

In the protocol a cost-effectiveness analysis was planned, but we will not conduct that analysis now. Therefore, we adjusted the secondary outcomes and removed all measures required for cost-effectiveness treatment, namely use of tocolysis, corticosteroids, magnesium-sulphate and maternal admission days.

Regarding the subgroup analyses, we removed the sub analysis concerning the 25th, 25th-50th, and above the 50th percentile of the cervical length distribution, because of limited clinical relevance and limited value in comparison with the predefined subgroup of cervical length ≤25mm and >25mm.

In this subgroup analysis of cervical length ≤25mm and >25mm (including an interaction term) on cervical length, next to assessing the primary outcome, in addition the following secondary outcomes will be assessed:

- Spontaneous PTB rate before 34 weeks of GA (n/N, %)
- Spontaneous PTB rate before 28 weeks of GA (n/N, %)

In addition, we will perform an exploratory analysis on (spontaneous) PTB rates < 24, < 28, <32, <34 and < 37 weeks for the subgroup of women with a cervical length ≤25mm and >25mm. We will also conduct an subgroup analysis of children born before or at 36 weeks or above 36 weeks. We will assess the primary outcome, spontaneous PTB before 34 and 28 weeks en time to delivery with a Kaplan Meier curve censored at 36 weeks.

Lastly, to investigate whether a margin can be found in the intensity of treatment, we will perform a sensitivity analysis of the per protocol population by looking at 60-100% PCD.

9. Presentation of study results

9.1 Recruitment

The recruitment of study participants will be presented using the CONSORT flow diagram. Exact details of screened patients cannot be given, since not all data have not been collected and digitalized yet.

9.2 Protocol violations

Severe protocol violations will be pointed out in the Consort Flow figure with reason of violation.

9.3 Baseline characterisations

The baseline characteristics will be presented for the total population as randomised (intention-to-treat), using the format of the mock table included below. Data will be presented using absolute numbers with percentages for discrete outcomes. Continuous outcomes will be presented as means with standard deviation, or medians with interquartile ranges.

	Pessary (n =)	Progesterone (n =)
Maternal age, years (mean, SD)	XX (xx)	XX (xx)
Body-mass index, kg/m^2 (mean, SD)	XX.X (xx)	XX.X (xx)

Education<br>Low*	NNN (%)	NNN (%)
Ethnicity		
White	NNN (%)	NNN (%)
Black	NNN (%)	NNN (%)
Middle Eastern	NNN (%)	NNN (%)
Asian	NNN (%)	NNN (%)
Other	NNN (%)	NNN (%)
Unknown	NNN (%)	NNN (%)
Current smoker	NNN (%)	NNN (%)
Uterus anomaly	NNN (%)	NNN (%)
Nulliparous	NNN (%)	NNN (%)
Previous preterm birth ($34^{+0} – 36^{+6}$)	NNN (%)	NNN (%)
History of cervical surgery (Conisation/LLETZ***)	NNN (%)	NNN (%)
History of curettage	NNN (%)	NNN (%)
Conception		
Pregnancy after IVF/ ICSI****	NNN (%)	NNN (%)
3 or more fetus	NNN (%)	NNN (%)
Monochorionic pregnancy	NNN (%)	NNN (%)
GA (weeks+days) at randomisation (median, IQR)	XX+X (XX+X - XX+X)	XX+X (XX+X - XX+X)
Cervical length at randomisation (mm) (mean, SD)	XX.X (xx)	XX.X (xx)
Cervical length range	NNN (%)	NNN (%)
0-15 mm	NNN (%)	NNN (%)
16-20 mm	NNN (%)	NNN (%)
21-25 mm	NNN (%)	NNN (%)
26-30 mm	NNN (%)	NNN (%)
31-37 mm	NNN (%)	NNN (%)
Funneling	NNN (%)	NNN (%)

* Primary school, prevocational secondary education (VMBO in Dutch)

** Senior general secondary education (HAVO in Dutch), pre-university secondary education (VWO in Dutch), secondary vocational education (MBO in Dutch), higher professional education (HBO in Dutch), and university education (WO in Dutch).

***LLETZ, large loop excision of the transformation zone

****ISCI, intracytoplasmic sperm injections, IVF, in vitro fertilization

Table 1: Baseline characteristics

9.4 Primary and secondary outcomes

The primary and secondary outcomes be presented for the total population as randomised (intention-to-treat) using the format of the mock table included below.

	Pessary<br>N=	Progesterone<br>N=	RR (95% CI)	P-value
**Primary outcome on child level**				
Composite adverse neonatal outcome (ITT), crude	*NNN (%)*	*NNN (%)*	*RR (95% CI)*	
Composite adverse neonatal outcome (ITT), adjusted for centre and mother	*NNN (%)*	*NNN (%)*	*RR (95% CI)*	

Table 2. Primary outcome: composite adverse neonatal outcome on child level

MATERNAL LEVEL				
	Pessary<br>N=	Progesterone<br>N=	RR (95% CI)	P-value
Composite adverse neonatal outcome (ITT), crude	*NNN (%)*	*NNN (%)*	*RR (95% CI)*	x.xx

Composite adverse neonatal outcome (ITT), adjusted for centre	*NNN (%)*	*NNN (%)*	*RR (95% CI)*	x.xx
Obstetric outcomes				
Preterm birth < 37 weeks	NNN (%)	NNN (%)	RR (95% CI)	x.xx
sPTB < 37 weeks	NNN (%)	NNN (%)	RR (95% CI)	x.xx
PTB < 34 weeks	NNN (%)	NNN (%)	RR (95% CI)	x.xx
sPTB < 34 weeks	NNN (%)	NNN (%)	RR (95% CI)	x.xx
PTB < 32 weeks	NNN (%)	NNN (%)	RR (95% CI)	x.xx
sPTB < 32 weeks	NNN (%)	NNN (%)	RR (95% CI)	x.xx
PTB < 28 weeks	NNN (%)	NNN (%)	RR (95% CI)	x.xx
sPTB < 28 weeks	NNN (%)	NNN (%)	RR (95% CI)	x.xx
Time to delivery (days), mean (SD)	XX.X (xx)	XX.X (xx)	RR (95% CI)	x.xx
PPROM*	NNN (%)	NNN (%)	RR (95% CI)	x.xx
Cerclages	NNN (%)	NNN (%)	RR (95% CI)	x.xx
Maternal outcomes				
Maternal mortality	NNN (%)	NNN (%)	RR (95% CI)	x.xx
Maternal morbidity				
Thromboembolic complication	NNN (%)	NNN (%)	RR (95% CI)	x.xx
Pre-eclampsia/HELLP**	NNN (%)	NNN (%)	RR (95% CI)	x.xx
Chorioamnionitis	NNN (%)	NNN (%)	RR (95% CI)	x.xx
Urinary tract infections	NNN (%)	NNN (%)	RR (95% CI)	x.xx
Genital tract infections	NNN (%)	NNN (%)	RR (95% CI)	x.xx
Pneumonia	NNN (%)	NNN (%)	RR (95% CI)	x.xx
Endometritis	NNN (%)	NNN (%)	RR (95% CI)	x.xx

CHILD LEVEL

	Pessary N=	Progesterone N=	RR (95% CI)	P-value
Composite adverse neonatal outcome (PP), crude	*NNN (%)*	*NNN (%)*	*RR (95% CI)*	x.xx
Neonatal outcomes				
Mode of delivery				
Vaginally	NNN (%)	NNN (%)	RR (95% CI)	x.xx
Caesarean section	NNN (%)	NNN (%)	RR (95% CI)	x.xx
Birth weight (g), mean (SD)	XX.X (xx)	XX.X (xx)	RR (95% CI)	x.xx
Birthweight <2500 gram (%)	NNN (%)	NNN (%)	RR (95% CI)	x.xx
Birthweight <1500 gram (%)	NNN (%)	NNN (%)	RR (95% CI)	x.xx
Neonatal diagnosis				
Patent ductus arteriosus	NNN (%)	NNN (%)	RR (95% CI)	x.xx
Treated seizures	NNN (%)	NNN (%)	RR (95% CI)	x.xx
Chronic Lung disease#	NNN (%)	NNN (%)	RR (95% CI)	x.xx
PVL > grade 1#	NNN (%)	NNN (%)	RR (95% CI)	x.xx
IVH grade III or IV#	NNN (%)	NNN (%)	RR (95% CI)	x.xx
NEC > stage 1#	NNN (%)	NNN (%)	RR (95% CI)	x.xx
ROP#	NNN (%)	NNN (%)	RR (95% CI)	x.xx
Culture proven sepsis (total)	NNN (%)	NNN (%)	RR (95% CI)	x.xx
< 72 hours after birth (early)	NNN (%)	NNN (%)	RR (95% CI)	x.xx
> 72 hours after birth (late)	NNN (%)	NNN (%)	RR (95% CI)	x.xx
Perinatal death	NNN (%)	NNN (%)	RR (95% CI)	x.xx
NICU admission (days)^, median (IQR)	NN (xx-xx)	NN (xx-xx)	RR (95% CI)	x.xx
Congenital abnormalities	NNN (%)	NNN (%)	RR (95% CI)	x.xx

*PPROM: premature rupture of membranes
**HELLP: haemolysis, elevated liver enzymes and low platelets
#Severe respiratory distress syndrome (RDS) or Bronchopulmonary Dysplasia (BPD); PVL: Periventricular leukomalacia; IVH: Intraventricular Haemorrhage; NEC: Necrotizing Enterocolitis; ROP: Retinopathy of prematurity
^NICU: neonatal intensive care unit

Table 3 – Secondary outcomes on maternal and child level

9.5. Subgroup analyses

	Pessary	Progesterone	Relative Risk (95% CI)	p-value interaction term
Cervical length				
Composite adverse perinatal outcome (primary outcome on child level)				
≤ 25 mm	N/ NN (%)	N/ NN (%)	RR (95% CI)	x.xx
> 25 mm	N/ NN (%)	N/ NN (%)	RR (95% CI)	
Spontaneous PTB <34 weeks (secondary outcome)				
≤ 25 mm	N/ NN (%)	N/ NN (%)	RR (95% CI)	x.xx
> 25 mm	N/ NN (%)	N/ NN (%)	RR (95% CI)	
Spontaneous PTB <28 weeks (secondary outcome)				
≤ 25 mm	N/ NN (%)	N/ NN (%)	RR (95% CI)	x.xx
> 25 mm	N/ NN (%)	N/ NN (%)	RR (95% CI)	

Table 3– Subgroup analyses on the primary outcome composite adverse perinatal outcome, sPTB < 34 and sPTB < 28 weeks

	Pessary	Progesterone	Relative Risk (95% CI)	p-value interaction term
Obstetric history				
Composite adverse perinatal outcome (primary outcome)				
Nulliparous women	N/ NN (%)	N/ NN (%)	RR (95% CI)	x.xx
Multiparous PTB $34^{+0} – 36^{+6}$ weeks	N/ NN (%)	N/ NN (%)	RR (95% CI)	
Multiparous term birth	N/ NN (%)	N/ NN (%)	RR (95% CI)	

Table 4– Subgroup analyses of parity on the primary outcome composite adverse perinatal outcome

	Pessary	Progesterone	Relative Risk (95% CI)	p-value interaction term
Chorionicity				
Composite adverse perinatal outcome (primary outcome on child level)				
Dichorionicity	N/ NN (%)	N/ NN (%)	RR (95% CI)	x.xx
Monochorionicity	N/ NN (%)	N/ NN (%)	RR (95% CI)	

Table 5 – Subgroup analyses of chorionicity on the primary outcome composite adverse perinatal outcome

	Pessary	Progesterone	Relative Risk (95% CI)	p-value interaction term
Number of foetuses				
Composite adverse perinatal outcome (primary outcome on child level)				
2 fetuses	N/ NN (%)	N/ NN (%)	RR (95% CI)	x.xx
3 or more fetuses	N/ NN (%)	N/ NN (%)	RR (95% CI)	

Table 6 – Subgroup analyses of number of fetuses on the primary outcome composite adverse perinatal outcome

9.6 Additional tables for supplement

Serious Adverse Events	Pessary (n=NNN)	Progesterone (n=NNN)	Relative Risk (95% CI)	p-value
Maternal death	NNN (%)	NNN (%)	RR (95% CI)	0.XX
Life threatening (at the time of event) to the mother	NNN (%)	NNN (%)	RR (95% CI)	0.XX
Hospitalization or prolongation for other than expected reason[#]	NNN (%)	NNN (%)	RR (95% CI)	0.XX
Persistent or significant disability or incapacity of the mother	NNN (%)	NNN (%)	RR (95% CI)	0.XX
Severe congenital anomaly or birth defect of the neonate	NNN (%)	NNN (%)	RR (95% CI)	0.XX
Any other important medical event[$]	NNN (%)	NNN (%)	RR (95% CI)	0.XX

[#]Requires hospitalization or prolongation of existing inpatients' hospitalization other than expected obstetric complications (such as threatened premature labour, admissions due to labour or scheduled
[$]Any other important medical event that may not result in death, be life threatening, or require hospitalization, may be considered a serious adverse experience when, based upon appropriate medical judgement, the event may jeopardize the subject or may require an intervention to prevent one of the outcomes listed above.

Table S1– Serious Adverse Events

	Pessary N= NN	Progesterone N=NN
Cervical length ≤25mm		
PTB < 37 weeks	NN (%)	NN (%)
sPTB < 37 weeks	NN (%)	NN (%)
PTB < 34 weeks	NN (%)	NN (%)
sPTB < 34 weeks	NN (%)	NN (%)
PTB < 32 weeks	NN (%)	NN (%)
sPTB < 32 weeks	NN (%)	NN (%)
PTB < 28 weeks	NN (%)	NN (%)
sPTB < 28 weeks	NN (%)	NN (%)
PTB < 24 weeks	NN (%)	NN (%)
sPTB < 24 weeks	NN (%)	NN (%)
Cervical length >25 mm		
PTB < 37 weeks	NN (%)	NN (%)
sPTB < 37 weeks	NN (%)	NN (%)
PTB < 34 weeks	NN (%)	NN (%)
sPTB < 34 weeks	NN (%)	NN (%)
PTB < 32 weeks	NN (%)	NN (%)
sPTB < 32 weeks	NN (%)	NN (%)
PTB < 28 weeks	NN (%)	NN (%)
sPTB < 28 weeks	NN (%)	NN (%)
PTB < 24 weeks	NN (%)	NN (%)
sPTB < 24 weeks	NN (%)	NN (%)

Table S2 – Exploratory analysis for cervical length ≤25mm and >25mm on the secondary outcomes (s)PTB <37, (s)PTB < 34, (s)PTB <32, (s)PTB <28 and (s)PTB < 24 weeks.

	Compliance threshold (% PDC)	Total N	Pessary n/N (%)	Progesterone n/N (%)	RR /mean difference (95% CI)	p-value
Composite adverse neonatal outcome, crude	100%	NN	NN / NN (XX%)	NN / NN (XX%)	X.X (XX to XX)	x.xx
	90%	NN	NN / NN (XX%)	NN / NN (XX%)	X.X (XX to XX)	x.xx
	80%	NN	NN / NN (XX%)	NN / NN (XX%)	X.X (XX to XX)	x.xx
	70%	NN	NN / NN (XX%)	NN / NN (XX%)	X.X (XX to XX)	x.xx
	60%	NN	NN / NN (XX%)	NN / NN (XX%)	X.X (XX to XX)	x.xx

PDC, proportion of days covered

Table S3 - Per protocol analyses for various compliance thresholds of PDC

	Pessary (n =)		Progesterone (n =)	
Allocated treatment initiated	NN (%)		NN (%)	
**Removal of pessary or stop progesterone**				
*According to study protocol^*	NN (%)		NN (%)	
Gestational age > 36 weeks	NN (%)		NN (%)	
Contractions or labour	NN (%)		NN (%)	
Other reasons, required delivery	NN (%)		NN (%)	
(P)PROM	NN (%)		NN (%)	
*Not according to study protocol^*	NN (%)		NN (%)	
	Stopped NN (%))	*Switched* NN (%)	*Stopped* NN (%)	*Switched* NN (%)
Discomfort	NN (%)	NN (%)	NN (%)	NN (%)
Excessive discharge	NN (%)	NN (%)	NN (%)	NN (%)
Patient preference	NN (%)	NN (%)	NN (%)	NN (%)
Pessary (re)placement failure	NN (%)	NN (%)	NA	NA
Vaginal blood loss	NN (%)	NN (%)	NN (%)	NN (%)
Compliance <100% PDC	NN (%)	NN (%)	NN (%)	NN (%)
Other	NN (%)	NN (%)	NN (%)	NN (%)
Cerclage	NN (%)	NN (%)	NN (%)	NN (%)
Total pessary replacements	NN		NA	
Failed	NN		NA	

^ If multiple reasons were given, one was chosen to be displayed in table
PDC, proportion of days covered; PPROM, premature preterm rupture of membranes

Table S4 – Details of intervention use: reasons for premature termination of allocated intervention

10. Figures

10.1 Flowchart of participants

The flow of study participants will be presented using the CONSORT flow-chart for clinical trial participants as shown below.

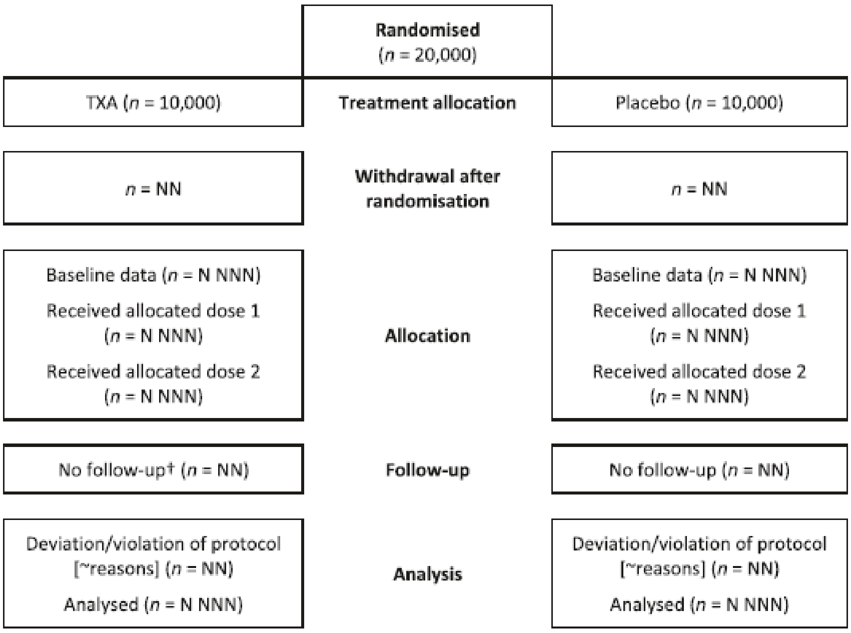

Fig. 1 Trial profile. †No follow-up relates to those patients where there is no information on the primary endpoint

10.2 Kaplan Meier curves for time to delivery

Kaplan-Meier plots will be constructed to illustrate the time to delivery from the day of randomisation in the intention to treat-population. The difference in gestational age at birth between the treatment groups will be tested using a log-rank test.
