# Supplementary material for: Cervical pessary versus vaginal progesterone in women with a multiple pregnancy and a short cervix: A randomised controlled trial
Source: PLoS Med. 2025 Nov 3;22(11):e1004586. doi: 10.1371/journal.pmed.1004586 (PMC12591417; doi:10.1371/journal.pmed.1004586)
Supplement: S4 File — (PDF) [file pmed.1004586.s004.pdf]

Academisch Medisch Centrum

Universiteit van Amsterdam

Aan de heer prof.dr. B.W.J. Mol
Verloskunde / gynaecologie
H4-256

Medisch Ethische Toetsingscommissie
E2-170
telefoon: 020 56 67389
fax: 020 56 69015

Amsterdam, 19 november 2013
ons kenmerk: 2013_019#B2013994
betreft: **Positief besluit**
NL42926.018.13
Pessary or progesterone to prevent preterm delivery in women with short cervical length. Quadruple P study

Geachte heer Mol,

De METC AMC heeft zich, op grond van artikel 2, lid 2, sub a van de Wet medisch-wetenschappelijk onderzoek met mensen (WMO) beraden over bovengenoemd onderzoeksdossier.
Wij delen u gaarne mee dat onze commissie
- tot oordelen bevoegd krachtens artikel 2, tweede lid, onder a, van de Wet medisch-wetenschappelijk onderzoek met mensen (WMO);
- werkzaam volgens de ICH-GCP richtlijnen;
- op grond van de haar voorgelegde stukken als hierna vermeld;
- gelet op artikel 3 van de WMO;
- gelet op artikel 5 en 6;
- vastgesteld hebbende dat voorzien is in de dekking van een aansprakelijkheidsverzekering als bedoeld in artikel 7, lid 9 van de WMO,

heeft besloten tot een positief oordeel over deze studie en de uitvoering daarvan in de volgende centra: het AMC.

Voorts hebben wij vastgesteld dat voor het onderzoek een verzekering is afgesloten conform de WMO door het AMC ten behoeve van proefpersonen van het AMC.

In de beoordeling betrokken documenten:
A1 aanbiedingsbrief d.d. 20 januari 2013
A1 aanbiedingsemail d.d. 28 januari 2013 1
A1 aanbiedingsemail d.d. 28 januari 2013 2
A1 aanbeidingsbrief d.d. 10 juli 2013
A1 aanbiedingsemail d.d. 15 juli 2013
A1 aanbiedingsbrief d.d. 2 september 2013, ongetekend
A1 aanbiedingsemail d.d. 3 september 2013
A1 aanbiedingsbrief d.d. 13 november 2013
A1 aanbiedingsemail d.d. 14 november 2013
A3 ontvangstbewijs EudraCT email d.d. 24 juni 2013
B1 ABR-formulier NL42926.018.13 versie 05 d.d. 13 november 2013
B2 AMC-appendix, getekend d.d. 12 juli 2013
B3 EudraCT aanvraag, getekend d.d. 11 juli 2013
C1 protocol versie 1.3 d.d. 13 november 2013 TC
D2 SPC utrogestan d.d. 2009
D2 EC certificaat d.d. 3 juli 2012
D2 SPC Progesterone 200mg d.d. december 1993
D2 SPC Utlogestan 200mg capsules d.d. februari 2006
D3 voorbeeldetiketten versie 1.2 d.d. 28 juni 2013

D4 certificaat MEDCERT d.d. 28 mei 2008
D4 certificaat No. 10610GB412120711 d.d. 11 juli 2012
D4 certificaat No. 1340GB417120704 d.d. 4 juli 2012
E1 E2 proefpersoneninformatie en toestemmingsverklaring versie 1.3 d.d. 13 november 2013 TC
I1 lijst deelnemende centra versie d.d. 11 juli 2013
K5 DSMB charter versie d.d. 10 juli 2013
K6 monitoring plan versie 1.1 d.d. 10 juli 2013

Het onderzoeksdossier, aan ons ter beoordeling voorgelegd op 28 januari 2013, is besproken in de vergadering van onze commissie van 7 februari 2013, en vervolgens aan de orde geweest in de vergadering van het dagelijks bestuur van onze commissie van 10 september 2013. In deze vergadering is besloten de reactie d.d. 3 september 2013 te bespreken in de vergadering van onze commissie van 19 september 2013, waarna de hoofdonderzoeker is uitgenodigd in de vergadering van onze commissie van 17 oktober 2013 een mondelinge toelichting te geven over de reguliere behandeling die patiënten krijgen buiten studieverband, omdat op grond van de inhoud van de proefpersoneninformatie kon worden geconcludeerd dat patiënten een bewezen effectieve behandeling zou worden onthouden. De vragen van de commissie zijn met de mondelinge toelichting voldoende beantwoord. De verdere afhandeling, namelijk in het protocol en in de proefpersoneninformatie laten opnemen dat patiënten buiten studieverband in overleg met hun behandelaar kunnen kiezen voor behandeling in plaats van reguliere controle, is gemandateerd aan de secretaris. Deze heeft geconstateerd dat met het voorleggen van de aangepaste stukken d.d. 14 november 2013 aan dit verzoek is voldaan.

De commissie heeft voorts vastgesteld dat het geneesmiddelenonderzoek betreft als bedoeld in artikel 13a van de WMO en dat tevens aan alle in artikel 13d van de WMO genoemde aanvullende voorwaarden is voldaan.

U dient onze commissie op de hoogte te stellen van de daadwerkelijke start van het onderzoek, van de (al dan niet voortijdige) beëindiging daarvan, en van tijdens de studie optredende onverwachte complicaties. Voorts dienen eventuele protocolwijzigingen ter beoordeling aan onze commissie te worden voorgelegd. U dient tevens ons jaarlijks een voortgangsrapportage betreffende de studie te doen toekomen, voor het eerst binnen een jaar na dagtekening van dit besluit.

Wij wijzen u erop dat op grond van artikel 23 van de Wet medisch-wetenschappelijk onderzoek met mensen degene wiens belang rechtstreeks bij een besluit van de MEC is betrokken, daartegen binnen zes weken na de dag waarop het besluit bekend is gemaakt, een administratief beroepschrift kan indienen bij de Centrale Commissie Mensgebonden Onderzoek. Een dergelijk administratief beroepschrift dient geadresseerd te worden aan: CCMO, Postbus 16302, 2500 BH Den Haag.

Tenslotte brengen wij onder uw aandacht dat dit besluit zijn geldigheid verliest als de studie niet binnen één jaar na dagtekening van deze brief is gestart. Voorts dient voor de uitvoering van geneesmiddelonderzoek naast een positief oordeel van onze commissie ook een verklaring van geen bezwaar van de CCMO als bevoegde instantie verkregen te zijn (zie voor de procedure de website van de CCMO).

Ten tijde van de beoordeling van dit project was de commissie als volgt samengesteld:

prof.dr. M.P.M. Burger	:	voorzitter, gynaecoloog
mw.drs. G.H.M. van Ammers	:	lid dat onderzoek beoordeelt vanuit de invalshoek van de proefpersoon
dr. A.J. Bredenoord	:	maag darm lever arts
dr. M.G.W. Dijkgraaf	:	plv. lid, methodoloog
mw. J.M.M. Dijkstra	:	lid dat onderzoek beoordeelt vanuit de invalshoek van de proefpersoon
prof.dr. R.C.M. Hennekam	;	hoogleraar kindergeneeskunde en klinische genetica
prof. dr H.A. Heij	:	hoogleraar kinderchirurgie
dr. M.T. Hilhorst	:	plv. lid, ethicus
prof.dr. J.J. Homan van der Heide	:	internist
dr. J.M.N.E. Jans	:	plv. lid, ethicus
dr. R.E. Jonkers	:	longarts/klinisch farmacoloog
mw.dr. E.M. Kemper	:	ziekenhuisapotheker, klinisch farmacoloog
dr. M.J.W. Koelemay	:	vaatchirurg
mw.dr. S.J. de Kort	:	plv. lid, medisch ethicus
prof.mr.dr. J. Legemaate	:	plv. lid, hoogleraar gezondheidsrecht
prof.dr. R.A.A. Mathôt	:	ziekenhuisapotheker, klinisch farmacoloog

dr. G.A. van Montfrans	:	internist
mw.dr. W.M.C. Mulder	:	plv. lid, klinisch farmacoloog
dr. P.J Nederkoorn	:	neuroloog
dr. M. Nieuwdorp	:	internist
mw.mr.dr. M.C. Ploem	:	gezondheidsjurist
dr. G. ter Riet	:	plv. lid methodoloog
prof.dr. A.J.P.M. Smout	:	hoogleraar gastroenterologie
mw.mr. L.M. Spittuler	:	lid dat onderzoek beoordeelt vanuit de invalshoek van de proefpersoon
dr. H.L. Tan	:	cardioloog
prof.dr. J.G.P. Tijssen	:	hoogleraar klinische epidemiologie van hart- en vaatziekten
prof.dr. M. Vermeulen	:	hoogleraar neurologie
mw.dr. A.M. Westermann	:	internist-oncoloog
prof.dr. D.L. Willems	:	hoogleraar medische ethiek
prof.dr. A.H. Zwinderman	:	plv. lid hoogleraar biostatistiek.

Voor de exacte samenstelling van de commissie tijdens de vergadering waarin het besluit is genomen, kunt u contact opnemen met het secretariaat van de commissie.

Met vriendelijke groet,
namens de Medisch Ethische Toetsingscommissie,

Mw. T. Groenveld,
ambtelijk secretaris

c.c. CCMO (pdf via TOL)
c.c. AMC Medical Research BV (pdf per e-mail) (+)
c.c. pdf per e-mail F.J.R. Hermans, info@studies-obsgyn.nl, S.M.S. Liem
c.c. CRU (pdf per e-mail)
c.c. apotheek (pdf per e-mail)

S1 Study Approval (English Translation)

Note to readers

The following is an English translation of the official approval letter issued in Dutch by the Medical Ethics Review Committee (METC) of the Academic Medical Center (AMC), Amsterdam, for the Quadruple P study. This translation is provided for submission to PLOS Medicine and faithfully reflects the content of the original Dutch document.

Ethical Study Approval Letter

Subject: Positive decision – Pessary or progesterone to prevent preterm delivery in women with short cervical length (Quadruple P study)
Reference number: NL42926.018.13 / 2013_019#B2013994
Date of decision: November 2013
Issued by: Medical Ethics Review Committee (METC) of the Academic Medical Center (AMC), Amsterdam, The Netherlands

Dear Dr. Mol,

The Medical Ethics Review Committee (METC) of the Academic Medical Center (AMC) has reviewed, in accordance with Article 2, paragraph 2, sub a, of the Medical Research Involving Human Subjects Act (WMO), the above-mentioned research file.

We hereby inform you that this Committee, being competent to assess such research under Article 2, paragraph 2, sub a, of the WMO, acting in accordance with the ICH-GCP guidelines and based on the submitted documentation listed below, having considered Articles 3, 5, 6, and 7 of the WMO, and having established that appropriate liability insurance is in place in accordance with Article 7, paragraph 9, of the WMO, has decided to issue a positive opinion on this study and its conduct at the following sites within the AMC.

Furthermore, the Committee has confirmed that insurance has been arranged in accordance with the WMO on behalf of the AMC for study participants recruited through the AMC.

Documents reviewed:
List of documents as in the original: submission letters, protocol versions, certificates, monitoring plan, informed consent materials, etc.

The research dossier was initially submitted for review on 28 January 2013 and discussed in Committee meetings on 7 February 2013, 10 September 2013, 19 September 2013, and 17 October 2013. Following these discussions and revisions to the participant information form and protocol, the Committee concluded that the information provided to participants sufficiently explained that patients outside the study would receive standard care, while study participants could receive one of the interventions under investigation. The Committee found the revised documents dated 14 November 2013 satisfactory and in compliance with the WMO.

The Committee has determined that this study qualifies as medical research involving medicinal products as defined in Article 13a of the WMO and that all additional requirements set forth in Article 13d of the WMO have been met.

You are requested to notify this Committee of the actual start date of the study, any (premature) termination, and any unexpected complications during the study. Protocol amendments must be submitted to the Committee for review. An annual progress report on the conduct of the study must also be submitted within one year of this decision.

We draw your attention to Article 23 of the WMO, which provides that any person directly involved in this decision may lodge an administrative appeal with the Central Committee on Research Involving Human Subjects (CCMO) within six weeks of the date of this notification.

Finally, please note that this approval expires if the study does not commence within one year of the date of this letter. Prior to study initiation, you must obtain a statement from the CCMO confirming that no objection has been raised against the study's conduct.

See original document for complete list of Medical Ethics Review Committee.

Sincerely,
on behalf of the Medical Ethics Review Committee (METC) of the AMC,

Mr. T. Groenveld
Administrative Secretary

cc:
- CCMO (via TOL, pdf)
- AMC Medical Research BV
- CRU
- Study investigators (via email)
- Hospital pharmacy (via email)
