# Supplementary material for: Cervical pessary versus vaginal progesterone in women with a multiple pregnancy and a short cervix: A randomised controlled trial
Source: PLoS Med. 2025 Nov 3;22(11):e1004586. doi: 10.1371/journal.pmed.1004586 (PMC12591417; doi:10.1371/journal.pmed.1004586)
Supplement: S5 File — (PDF) [file pmed.1004586.s005.pdf]

S1 (Supplement F) VOC declaration of intent regarding incubator infants

ZonMW
Programma Goed Gebruik Geneesmiddelen
Laan van Nieuw Oost Indië 334
2593 CE Den Haag

Leidschendam 9 september 2012
Referentie: gj\12VOC019
Betreft: ondersteuning subsidieaanvraag 80-83600-98-10119, the Quadruple P trial.

Geachte programmaleider,

Op verzoek van de indieners van de bovenvermelde subsidieaanvraag heeft het bestuur van de Vereniging van Ouders van Couveusekinderen (VOC) kennisgenomen van de onderzoek aanvraag van Dr. Boormans en medeonderzoekers: Pessary or Progesterone to Prevent Preterm delivery in women with short cervical length (Quadruple P Trial).

De VOC houdt zich bezig met de belangen van (aankomende) ouders in de volle breedte: préconceptioneel, preventie, neonatologische zorg en nazorg/follow-up. In de afgelopen jaren is er ook bij ons, vertegenwoordigers van prematuur en/of dysmatuur geboren kinderen, groeiende aandacht voor préconceptionele advisering en richtlijnen ter preventie van vroeggeboorte bij zwangere vrouwen met een (hoog) risico op vroeggeboorte.

De VOC is aangesloten en bestuurlijk betrokken bij de koepel van Europese ouderorganisaties, de EFCNI (European Foundation for the Care of Newborn Infants). Eind vorig jaar deed de EFCNI een oproep aan de leden van het Europees Parlement. In deze zogeheten Call-to-Action (http://www.efcni.org/index.php?id=1890), wordt ook aangedrongen op nationale richtlijnen voor preventieve maatregelen en préconceptionele advisering. De Call-to-Action heeft recentelijk ook geleid tot initiatieven richting de Nederlandse politiek.

Wij vinden het onderwerp van het bij Zon Mw ingediende onderzoeksvoorstel dan ook van buitengewoon belang, aangezien het een patiëntencategorie (zwangere vrouwen met verhoogd risico
op vroeggeboorte) betreft waar tot op heden geen effectieve therapie bestaat die de thans zeer slechte
prognose significant kan verbeteren.

Wij ondersteunen de aanvraag daarom van harte.

Namens het VOC bestuur,

Hoogachtend,

Dr. G.J. van Steenbrugge,
Directeur

S1 Declaration of Intent (English Translation)

Note to readers
This English translation of the original Dutch letter of intent from the Vereniging van Ouders van Couveusekinderen (VOC) is provided as supplementary material to ensure transparency and accessibility for international readers. The original document was included to demonstrate patient organization support for the Quadruple P Trial.

VOC Declaration of Intent regarding Incubator Infants

ZonMW
Program "Good Use of Medicines"
Laan van Nieuw Oost-Indië 334
2593 CE The Hague

Leidschendam, 9 September 2012
Reference: gj\12VOC019
Subject: Support for grant application 80-83600-98-10119, The Quadruple P Trial

Dear Program Director,

At the request of the applicants of the above-mentioned grant proposal, the board of the Association of Parents of Premature Infants (VOC) has reviewed the research proposal submitted by Dr. Boormans and co-investigators: Pessary or Progesterone to Prevent Preterm Delivery in Women with Short Cervical Length (Quadruple P Trial).

The VOC is dedicated to representing the interests of (expectant) parents across the full spectrum of care: preconception, prevention, neonatal care, and postnatal follow-up. In recent years, among our members, representing parents of preterm and/or growth-restricted infants, there has been increasing attention to preconception counseling and guidelines aimed at preventing preterm birth among pregnant women at (high) risk of such outcomes.

The VOC is a member of and actively involved in the European umbrella organization EFCNI (European Foundation for the Care of Newborn Infants). Late last year, the EFCNI issued a call to Members of the European Parliament. In this so-called Call to Action (http://www.efcni.org/index.php?id=1890), the need for national guidelines on preventive measures and preconception counseling was emphasized. This Call to Action has recently also led to initiatives within Dutch politics.

We therefore consider the subject of the research proposal submitted to ZonMw to be of exceptional importance, as it concerns a patient population (pregnant women at

increased risk of preterm birth) for whom no effective therapy currently exists that can significantly improve the currently very poor prognosis.

We therefore wholeheartedly support this grant application.

On behalf of the VOC Board,

Yours sincerely,

Dr. G.J. van Steenbrugge
Director
