# Supplementary material for: Cervical pessary versus vaginal progesterone in women with a multiple pregnancy and a short cervix: A randomised controlled trial
Source: PLoS Med. 2025 Nov 3;22(11):e1004586. doi: 10.1371/journal.pmed.1004586 (PMC12591417; doi:10.1371/journal.pmed.1004586)
Supplement: Attachment — Submitted filename: QP_Multiples_Letter of Response_final.docx [file pmed.1004586.s009.docx]

***Louise Gaynor-Brook, MBBS PhD***

***Senior Editor***

***PLOS Medicine***

Date: 13-jul-25

Our reference: PMEDICINE-D-25-00762R1

Title: **Cervical pessary versus vaginal progesterone in women with a multiple pregnancy and a short cervix: a cluster randomised controlled trial**

**Subject: Re-submission of manuscript**

Dear Louise Gaynor-Brook,

We appreciate the opportunity to revise this manuscript based on the valuable comments provided by the reviewers and the members of the editorial committee. We would like to thank the reviewers and editors for their helpful comments and feedback on our manuscript.

We have made a concerted effort to address each of the comments and organized them as tables in the sequence in which they have been raised. We have attached a copy of the revised manuscript with tracked changes, as well as a clean copy.

We would also like to apologize for submitting our revisions three days after the original deadline. On the day of the deadline, we realized that one final point required clarification with our statistician. We were able to resolve and integrate this issue into the manuscript in the following days. As mentioned in our email on the deadline date, we had requested a short extension until July 15th, but we are pleased to have completed the revisions earlier than anticipated. We hope that, despite this brief delay, our revised submission will still be taken into consideration.

We hope that our revisions will be in line with the expectations set out by the reviewers and the editors.

Thank you for your consideration of this article. We look forward to your response and decision on the manuscript.

Sincerely,

On behalf of the authors,

Charlotte van Dijk

Department of Obstetrics and Gynaecology

Amsterdam UMC, Amsterdam,

The Netherlands

**Reviewer Comments**

**Reviewer 1**

Reviewer 1; Comment 1

| A. Reviewer | The following suggestions are intended to further strengthen the study and are minor in nature:  1. Line 131- "main" would be better worded "primary"  2. Line 142- "case" should be "cases"  3. Line 161-Individuals do not need to be listed.  4. Line 196-A start date of the second randomization program but not the first.  5. Line 205 - If possible numerically what the words "most of whom" means.  6. Line 280- "to the other" either delete the word the or specify what the other means.  7. Line 425- Consider changing malacia to leukomalacia.  8. Line 604- The groups need to be labelled at the top. |
| --- | --- |
| B. Response | Thank you for your suggestions to further strengthen this study. We have incorporated them all in the manuscript.  Extra response to comment 5.  Unfortunately, the exact number is not known, but they were all affiliated with medical centers that had previously participated in the ProTwin trial and/ or in the Quadruple P singletons trial. |
| C. Changes made | 1. Line 131: ‘main’ changed into ‘primary’  2. Line 143: ‘case’ changed into ‘cases’  3. Line 163-168: individual board members are deleted  4. Line 207: added ‘initially’ to indicate the starting point when using ALEA  5. Line 216: ‘most’ changed into ‘the majority’  6. Line 302: deleted ‘to the other’  7. Line 453: changed ‘malacia’ into ‘leukomalacia’  8. Line 630: apologies, the heading of table 1 seems to be deleted during the submission, we have adjusted the table. |
| D. Location of changes | See above |

**Reviewer 2**

Reviewer 2; Comment 1

| A. Reviewer | Given that mothers were randomised but the primary outcome was measured on the infant level, this is a cluster randomised trial. Please ensure that reporting aligns with the CONSORT extension for cluster randomised trials. |
| --- | --- |
| B. Response | Thank you for your suggestion, we would like to clarify this important point.  Although outcomes are indeed also measured at the infant level, we do not interpret this study as a classic cluster randomised trial. In our design, randomisation occurred at the level of the individual pregnant woman. As described in the protocol, a single trial framework was established for all eligible women with either singleton or multiple pregnancies. These women were individually randomised to receive one of the interventions, and both mothers and fetuses, were followed during the trial and analysed accordingly. In addition to infant-level outcomes, our study also includes maternal outcomes, which are analysed at the level of the individual woman.  Although we acknowledge that in case of the multiple pregnancies a kind of clustered randomization has taken place, we could not establish the number of clusters involved, as we did not know how many women with a multiple pregnancy would be participating in this trial. Therefore, an associated sample size calculation with a prespecified number of clusters, for example, could not be executed.  In the subgroup of women with multiple pregnancies, we acknowledged that outcomes at the infant level (e.g. gestational age at birth, neonatal morbidity) may not be independent due to the shared intrauterine environment and maternal treatment allocation. Therefore, appropriate statistical methods (generalised estimating equations to account for clustering) were used to correct for possible correlation.  In summary, while we have adjusted for possible correlation between siblings in the analysis of infant outcomes, as of the combined trial design and type of interventions we believe that adherence to the standard CONSORT checklist for randomised trials remains the most appropriate for our study.  Nevertheless, for clarification we added more information on the trial design, randomization process and selection in the method section. |
| C. Changes made | Methods:  Line 204: After providing written informed consent, pregnant individuals were randomized to receive either a cervical pessary or vaginal progesterone in a 1 to 1 ratio…  Line 207: Also, the unborn multiples were automatically assigned to the randomization group of their mother. |
| D. Location of changes | Lines 204-205  Lines 207-208 |

Reviewer 2; Comment 2

| A. Reviewer | It is stated that this trial was stopped for futility, but there doesn't seem to be any allowance for that in the interim analysis section of the SAP. It is stated in the SAP that "there were no stopping rules based on the statistical significance of the effect of the treatment". How then was the trial stopped for futility? Please provide further details. |
| --- | --- |
| B. Response | You raise a pertinent issue, it touches on a matter that was also the subject of considerable internal discussion within our research group.  As outlined in the manuscript, interim analyses were conducted by an independent Data and Safety Monitoring Committee (DSMC). According to the Protocol, no formal stopping rules were defined based on statistical significance or futility. However, during the final interim analysis, the DSMC judged, based on the observed data (of 237 participants), a trend favouring vaginal progesterone, that continuing recruitment was no longer scientifically justified. They expected a very low probability that the cervical pessary would demonstrate superiority if the study were to be completed as planned. On that basis, the DSMC advised immediate termination of the trial.  Upon receiving this recommendation, we formally raised objections, as the original DSMC charter explicitly stated that the trial should not be stopped for futility alone. However, the DSMC stood by its recommendation, prioritizing scientific and ethical considerations over strict adherence to the initial agreement. The matter was escalated to the Medical Ethics Review Committee (METC) of the Amsterdam UMC, which appointed an independent panel to assess the situation. After reviewing the data and DSMC rationale, the METC supported the recommendation to halt the study. As a result, the trial was halted on 08 September 2023 after recruitment of 277 participants.  We acknowledge that this course of action deviated from the original protocol. However, given the oversight by both the DSMC and the independent ethics committee, we believe that the decision to stop the trial was ethically and scientifically sound, even though it was not based on pre-specified statistical stopping rules. |
| C. Changes made | To provide more details of these decisions, we have added the following sentence: “Although no formal futility stopping rules based on the statistical significance of the effect of the treatment were prespecified in the statistical analysis plan, the DSMC considered continued recruitment scientifically unjustified based on the observed data. Following an independent review by the METC of the Amsterdam UMC, it was concluded that the DSMC’s advice should be followed, leading to early termination of the trial, on the 8^th^ of September 2023. Follow-up ended in March 2024.” |
| D. Location of changes | Lines 331-335 |

Reviewer 2; Comment 3

| A. Reviewer | Was the sample size calculation based on the proportion of mothers who would experience adverse outcomes, or the number of babies who would experience adverse outcomes? If 332 participants is the number of mothers, then please clarify this. If the calculation was based on the number of babies, then the clustering of babies born to the same mother (i.e. multiple births) should have been accounted for. |
| --- | --- |
| B. Response | Thank you for this important question. The sample size calculation was based on the proportion of babies experiencing adverse perinatal outcomes (i.e., the primary outcome was defined at the neonatal level). However, the unit of randomization was the mother, and the sample size calculation was therefore performed in terms of 332 pregnant women (participants), 166 per arm.  We acknowledge that in twin/multiple pregnancies, outcomes are clustered within the same mother. However, this clustering was not explicitly adjusted for in the sample size calculation. This decision was based on precedent from a previous trial in a similar population, namely the ProTWIN study [ref 7], which also calculated sample size based on neonatal outcomes without adjusting for clustering due to the limited degree of intra-cluster correlation observed in that study.  Nonetheless, in the statistical analysis, the potential correlation between multiples was accounted for using generalized estimating equations (GEE), as described in the methods section. |
| C. Changes made | To specify on these calculations, we have adjusted the paragraph on the sample size calculation to specify more precisely and added an explanation for not clustering in the discussion:  Added to the description of the sample size:  Clustering of outcomes within multiple pairs was not accounted for. |
| D. Location of changes | Line 267 |

Reviewer 2; Comment 4

| A. Reviewer | Please provide more details regarding the randomisation. Were permuted blocks used? Was randomisation stratified by any variables? |
| --- | --- |
| B. Response | The randomisation was only stratified per centre and permuted variable block size of 2 and 4 were used. |
| C. Changes made | In order to clarify, this information on the block size was added in the method section. |
| D. Location of changes | Lines 205-207 |

Reviewer 2; Comment 5

| A. Reviewer | I was glad to see that baby-level outcomes were analysed by fitting a marginal model via generalised estimating equations to account for clustering. However, I think how this approach is described can be clarified in the Statistical analysis section. For example "Child-level outcomes were analysed via a marginal model fitted using generalised estimating equations, with an exchangeable working correlation matrix to account for clustering of multiples within mothers". Please note that the assumed working correlation matrix structure needs to be specified - was an exchangeable working correlation matrix assumed? |
| --- | --- |
| B. Response | Thank you for your comment. We used the robust estimator as covariance matrix and an independent working correlation matrix. The latter because there was no constant correlation between all pairs within the groups concerning the composite outcome. For example, the development of necrotising enterocolitis (NEC) in one twin during the neonatal period does not necessarily mean the other twin will also develop this condition. |
| C. Changes made | We added this information in the Method section. |
| D. Location of changes | Lines 273-274 |

Reviewer 2; Comment 6

| A. Reviewer | It is stated that "a sensitivity analysis with a mixed model using a random effect for the centre was conducted, allowing the extrapolation of outcomes to other centres". However, a mixed model with random effects for centre would estimate a centre-specific effect, so it is unclear how this sensitivity analysis achieved the aim of allowing extrapolation to other centres. Please clarify - were the random effects marginalised over? How was clustering by mother accounted for in this analysis? |
| --- | --- |
| B. Response | We appreciate the opportunity to clarify. We wrongly stated that the aim was to extrapolate to other centres. Our intention was to check the robustness of our primary model in which a marginal Generalized Estimated Equations model with centre as a fixed effect was used. In the mixed model both centre and mother were included as random effects.  We have clarified this point in the revised methods section and also revised the results section to clarify that the variation across centres was comparable, supporting the robustness of the primary analysis. |
| C. Changes made | Adjusted sentences:  Statistical analysis:  To ensure robustness of our primary analysis, a sensitivity analysis using a mixed-effects model including random effects for centre and mother was conducted.  Primary outcome:  A sensitivity analysis on the variability across centres was comparable (p = 0.48), supporting the robustness and generalizability of the primary outcome. |
| D. Location of changes | Line 277-280  Line 356-358 |

Reviewer 2; Comment 7

| A. Reviewer | The description of the analysis on line 268 ("At the neonatal level…") does not seem to describe any different analysis to that described above. What is the difference? (Please note that the working correlation matrix and link function also needs to be specified for this analysis). |
| --- | --- |
| B. Response | Thank you for pointing this out, there is no difference. We have made a clarification in the methods section. |
| C. Changes made | We have added: “comparable to the primary outcome” |
| D. Location of changes | Line 287 |

Reviewer 2; Comment 8

| A. Reviewer | It is stated that continuous outcomes were analysed via t-tests etc. However - the clustering of outcomes within mothers would need to be accounted for in this analysis too, if the outcomes are measured on the infant level. Marginal models fit via GEE or conditional models with random effects for mother should be fit to analyse these outcomes. |
| --- | --- |
| B. Response | We agree that these continuous outcomes should be analyzed taking into account the clustering within the mother. |
| C. Changes made | We changed the information on the analysis of continuous outcomes in the Method section in the following:  “…and continuous outcomes were analysed using GEE with a linear distribution and identity link with mean differences and the corresponding 95% confidence intervals along with the p-value.^28^ “ |
| D. Location of changes | Lines 288-294 |

Reviewer 2; Comment 9

| A. Reviewer | A per-protocol analysis is described - however, such an analysis compares groups based on post-randomisation variables and thus is subject to confounding and not an appropriate method for comparing groups. More appropriate methods of analysis would be to estimate complier average causal effects. For more information and more appropriate analysis techniques to be applied please see [https://www.sciencedirect.com/science/article/pii/S1836955321000059?via%3Dihub](%20https://www.sciencedirect.com/science/article/pii/S1836955321000059?via%3Dihub%20) (for example - there are plenty of other papers on this). The switching of treatments is an intercurrent event, and requires careful consideration and analysis: please see the ICH E9 statistical principles for clinical trials scientific guideline and the primer available at [https://eur04.safelinks.protection.outlook.com/?url=https%3A%2F%2Fdoi.org%2F10.1136%2Fbmj-2023- 076316&data=05%7C02%7Cc.e.vandijk%40amsterdamumc.nl%7Cf99750b9a0a64dbf2aac08ddb0387a38%7C68dfab1a11bb4cc6beb528d756984fb6%7C0%7C0%7C638860478805232308%7CUnknown%7CTWFpbGZsb3d8eyJFbXB0eU1hcGkiOnRydWUsIlYiOiIwLjAuMDAwMCIsIlAiOiJXaW4zMiIsIkFOIjoiTWFpbCIsIldUIjoyfQ%3D%3D%7C0%7C%7C%7C&sdata=KrqGSCGSQR%2F37ahLFKW%2BX2sZ9zIq7RPj5WwOR%2F5SrrE%3D&reserved=0](https://eur04.safelinks.protection.outlook.com/?url=https%3A%2F%2Fdoi.org%2F10.1136%2Fbmj-2023-%20076316&data=05%7C02%7Cc.e.vandijk%40amsterdamumc.nl%7Cf99750b9a0a64dbf2aac08ddb0387a38%7C68dfab1a11bb4cc6beb528d756984fb6%7C0%7C0%7C638860478805232308%7CUnknown%7CTWFpbGZsb3d8eyJFbXB0eU1hcGkiOnRydWUsIlYiOiIwLjAuMDAwMCIsIlAiOiJXaW4zMiIsIkFOIjoiTWFpbCIsIldUIjoyfQ%3D%3D%7C0%7C%7C%7C&sdata=KrqGSCGSQR%2F37ahLFKW%2BX2sZ9zIq7RPj5WwOR%2F5SrrE%3D&reserved=0). |
| --- | --- |
| B. Response | We acknowledge that the per protocol analysis groups focuses on data from participants who strictly adhered to the study protocol, and therefore bias could be introduced when events of exclusion happened after randomization. However, it was our intention to use the per protocol as a secondary analysis, checking the robustness of the primary intention-to-treat analysis. We followed the ICH-9 guideline for the selection and interpretation of the per protocol analysis. |
| C. changes made | No changes made |
| D. Location of changes | N.A. |

Reviewer 2; Comment 10

| A. Reviewer | A few participants were inappropriately randomised - and it seems that these patients were all excluded from analyses. Whether this is the most appropriate course of action is questionable - as described in PMID: 26033877, application of the intention-to-treat principle would mean that these patients are included in analyses. Please provide results of analyses including data from these patients as a sensitivity analysis. |
| --- | --- |
| B. Response | We fully acknowledge the importance of the intention-to-treat principle. However, in this particular case, the exclusion of a small number of inappropriately randomized participants was not a methodological decision but a legal and ethical requirement.  Specifically, for these participants, no written informed consent was available due to procedural errors during enrolment. In accordance with the General Data Protection Regulation (GDPR) and Good Clinical Practice (GCP) standards, the Amsterdam UMC Clinical Research Unit (CRU) concluded in consultation with the MEC of the Amsterdam UMC that retention and use of data from these individuals was not permitted. As such, all data for these participants were permanently deleted and are no longer accessible to the research team.  Consequently, it is not possible for us to include these participants in any sensitivity analyses or provide further details. |
| C. Changes made | None |
| D. Location of changes | N.A. |

Reviewer 2; Comment 11

| A. Reviewer | As stated in the CONSORT statement, hypothesis tests for baseline characteristics should not be conducted. Please remove the mention of the p-value in Table 1. |
| --- | --- |
| B. Response | It was ill-considered of us to include the p-values in Table 1. |
| C. Changes made | We have removed the p-values in Table 1. |
| D. Location of changes | Table 1, line 632 |

Reviewer 2; Comment 12

| A. Reviewer | Please include the number of mothers in each group at the top of Table 1 and the names of the groups. |
| --- | --- |
| B. Response | Apologies, the heading of table 1 seems to be deleted during the submission. |
| C. Changes made | We have adjusted the table and added the number of mothers and treatment modality. |
| D. Location of changes | Table 1, line 632 |

**Reviewer 3**

Reviewer 3; Comment 1

| A. Reviewer | Line 61 - not necessary to include limitations sentence in abstract |
| --- | --- |
| B. Response | According to the PLOS Medicine submission guidelines, it is mandatory to include the main limitations of the study in the Abstract. For this reason, we have included a concise statement describing the study's key limitations, in compliance with the journal’s requirements (see: *PLOS Medicine Submission Guidelines*). |
| C. Changes made | None |
| D. Location of changes | N.A. |

Reviewer 3; Comment 2

| A. Reviewer | Line 132 - WHO definition of extremely preterm birth is <28 weeks, very preterm is <32 weeks, please modify this sentence to ensure correct definitions. |
| --- | --- |
| B. Response | Thank you for this sharp comment, we have adjusted the first sentence to ensure correct definitions according the WHO definition. |
| C. Changes made | Sentence adjusted to: “The primary challenge in multiple pregnancies is the prevention of extremely or very preterm birth (PTB), defined as delivery before 28 and 32 weeks’ gestation respectively, … “ |
| D. Location of changes | Lines 131-132 |

Reviewer 3; Comment 3

| A. Reviewer | Line 143 - Ref 18 (EPPPIC) did not support progesterone use in multiples (RR 1.01). Please comment. |
| --- | --- |
| B. Response | Thank you for this comment. We agree that the EPPPIC meta-analysis (Ref 18) did not support the use of progesterone in multiple pregnancies, reporting a relative risk of 1.01, indicating no benefit. However, the systematic review and meta-analysis by Conde-Agudelo et al. (Ref 1) reported a significant reduction in the risk of preterm birth and neonatal morbidity and mortality with vaginal progesterone in women with a twin pregnancy and a short cervix (RR 0.58; 95% CI: 0.38–0.87). Both references are included to reflect the differing evidence in the literature and to highlight the clinical uncertainty that justified the current trial. |
| C. Changes made | None |
| D. Location of changes | N.A. |

Reviewer 3; Comment 4

| A. Reviewer | Line 181 - why do you use 38mm as a cut off, was it to be consistent with ProTwin Trial? I am interested to know how many multiples were not eligible for the study as in my experience, the vast majority will have cervical lengths below 38mm and not be at risk for preterm birth. My concern is that by including and treating the vast majority of multiples you are muddying the benefit of these therapies for those multiples actually at risk. |
| --- | --- |
| B. Response | Thank you for your comment. As described in our published protocol, the 38 mm cervical length cutoff was selected based on findings from the ProTwin trial, conducted in a comparable Dutch population. In that trial, although there was no overall benefit of pessary in unselected twin pregnancies, a significant reduction in preterm birth before 32 weeks was observed in the subgroup of women with a cervical length below the 25th percentile (38 mm) (11% vs. 25%; RR 0.44, 95% CI 0.20–0.98). Importantly, in this same subgroup, the rate of adverse perinatal outcome was substantially lower with pessary treatment (7% vs. 30%; RR 0.23, 95% CI 0.09–0.60) (Ref 19). Therefore, we used 38 mm as the inclusion threshold in our study to target the subgroup most likely to benefit, based on prior evidence.  Unfortunately, we were not able to collect reliable data on how many women were excluded due to cervical length ≥38 mm. Measuring cervical length in twin pregnancies is standard practice in the Netherlands; however, detailed data on the total screened population were not systematically documented.  For context, prior studies in similar populations (e.g., Hermans et al., *Am J Obstet Gynecol* 2018) reported a median cervical length of 43 mm in twin pregnancies in the Dutch population, with 38 mm representing the 25th percentile, which supports the appropriateness of this cutoff for identifying those at higher risk for preterm birth (positive likelihood for sPTB before 34 weeks of 2.4 (95% CI 1.7-3.2)). |
| C. Changes made | To clarify our cutoff value of 38mm for a short cervix, we have added the following sentence: “A cutoff of 38 mm was chosen as it was associated with a treatment benefit of a pessary in a comparable Dutch population of women with a twin pregnancy at higher risk of preterm birth.” |
| D. Location of changes | Lines 184-186 |

Reviewer 3; Comment 5

| A. Reviewer | You have excluded women with a cerclage, how common is this practice in your country and what are the indications for cerclage in multiples in your units? Does this mean that the most at risk women (ie. this with CL <15mm) will have a cerclage and therefore, be excluded from this study? |
| --- | --- |
| B. Response | Thank you for this important comment. In the Netherlands, cerclage is generally not performed in women with multiple pregnancies, as it is not part of standard clinical practice, especially during the study phase. In the last 2 years, it has become a more common practice, although we do not have precise national data on the overall number of procedures performed. This means that only a very small number of women with a cervical length <15 mm would have been excluded, as cerclage in multiples remains uncommon outside this setting.  We also note that the question of whether cerclage may benefit women with a twin pregnancy and a short cervix is currently being investigated in a Dutch multicenter randomized controlled trial – the TWIN-Cerclage studies (van Gils et al., *BMJ Open* 2024;14(5):e081561) – which may help define the role of cerclage in this population. This is mentioned at the end of the discussion. |
| C. changes made | None |
| D. Location of changes | N.A. |

Reviewer 3; Comment 6

| A. Reviewer | Methods: Why did you choose to stratify based on centre? |
| --- | --- |
| B. Response | Thank you for your question. Randomization was stratified by centre to prevent imbalances between treatment groups in aspects of maternal or neonatal care that may vary across centres, thereby reducing the risk of confounding due to centre-specific practices. |
| C. Changes made | We have added the following explanation in the methods section: “..to reduce the risk of confounding due to centre-specific practices.” |
| D. Location of changes | Lines 206-207 |

Reviewer 3; Comment 7

| A. Reviewer | Line 285 - I thought the exclusion criteria was no previous PTB <34 weeks? Please clarify your pre-defined sub group analysis and remove multiparous with prior delivery <34 weeks. |
| --- | --- |
| B. Response | Thank you for your comment. There may have been a misunderstanding. As per our protocol, women with a history of spontaneous preterm birth before 34 weeks of gestation were not eligible and were not included.  The predefined subgroup analysis compared nulliparous women, women with no history of spontaneous preterm birth, and those with a history of spontaneous preterm birth between 34 and 37 weeks gestation. Although rare, some women in the study had a previous spontaneous preterm birth between 34–37 weeks of gestation, and this group was included as a separate category in the subgroup analysis to explore whether late spontaneous preterm birth history influenced treatment effect. |
| C. Changes made | None |
| D. Location of changes | N.A. |

Reviewer 3; Comment 8

| A. Reviewer | Line 370 - can you comment on why the nulliparous women may show benefit of progesterone (cf pessary), compared with multiparous women? |
| --- | --- |
| B. Response | A very interesting question, of which the true answer is not known, as preterm birth is still covered in mysteries. Nulliparity is a known risk factor for spontaneous preterm birth. This group not only represents a substantial proportion of the overall pregnant population, but also contributes significantly to the total number of women at increased risk. In nulliparous women, there is a higher likelihood that cervical insufficiency may play a role in the risk of preterm birth, as cervical competence has not yet been demonstrated. In contrast, multiparous women have previously carried a pregnancy to a viable gestational age, which may indicate a more resilient cervix. Among women with a history of late preterm birth (34–37 weeks), it is hypothesized that cervical insufficiency may not have been the primary cause of preterm delivery. Since both progesterone and pessary appear to be most effective in women with short cervical length (≤25 mm), a potential treatment effect may be more apparent in nulliparous women, in whom cervical weakness is more likely to contribute to the risk profile. |
| C. Changes made | None |
| D. Location of changes | N.A. |

Reviewer 3; Comment 9

| A. Reviewer | Table 1 - no pessary and progesterone heading row, please add. |
| --- | --- |
| B. Response | Apologies, the heading of table 1 seems to be deleted during the submission. |
| C. Changes made | We have adjusted the table and added the number of mothers and treatment modality. |
| D. Location of changes | Table 1, line 632 |

**Reviewer 4**

Reviewer 4; Comment 1

| A. Reviewer | The primary outcome is a composite of adverse perinatal outcomes. On page 9 these are listed as PVL>1, severe RDS/BPD, IVH ¾, NEC, sepsis, stillbirth, death before discharge. On page 23 the composite results are listed and are adjusted - but it is not clear what is actually in the composite and which are the primary drivers.  Is perinatal death both stillbirth and death before discharge or is it the typical definition of death between 22wks and 7 days of life? |
| --- | --- |
| B. Response | Thank you for pointing out this uncertainty. We have indicated in table 4 which components are part of the composite outcome.  In this study, perinatal death is defined as death from gestational age of randomization to 10 weeks after the estimated due date. |
| C. Changes made | An # is placed in Table 4 behind the components of the composite outcome.  A definition of perinatal death is added. |
| D. Location of changes | Page 23, Table 4  Lines 237, 244-245 |

Reviewer 4; Comment 2

| A. Reviewer | Can the authors include in the table the composite and then below the composite the components of the composite? |
| --- | --- |
| B. Response | As mentioned above, in Table 4 we have indicated which components are part of the composite outcome. |
| C. Changes made | An # is placed in Table 4 behind the components of the composite outcome. |
| D. Location of changes | Page 23, Table 4 |

Reviewer 4; Comment 3

| A. Reviewer | Is death of one twin considered differently than death of both twins (or triplets)? |
| --- | --- |
| B. Response | No, no difference is made between death of one twin compared to death of both twins (or triplets). |
| C. Changes made | None |
| D. Location of changes | N.A. |

Reviewer 4; Comment 4

| A. Reviewer | Why was <38mm considered a short cervix in this population? |
| --- | --- |
| B. Response | Thank you for your comment. Reviewer 3 also commented on this cutoff value (comment 4). Therefore, we would like to refer you to our response on comment 4 of reviewer 3. |
| C. Changes made | To clarify our cutoff value of 38mm for a short cervix, we have added the following sentence: “A cutoff of 38 mm was chosen as it was associated with a treatment benefit of a pessary in a comparable Dutch population of women with a twin pregnancy at higher risk of preterm birth.” |
| D. Location of changes | Lines 184-186 |

Reviewer 4; Comment 5

| A. Reviewer | Why was prior sPTB <34 weeks an exclusion? |
| --- | --- |
| B. Response | Thank you for this question. According to the national prevention of preterm birth protocol in The Netherlands, women with a prior spontaneous preterm birth before 34 weeks of gestation are offered additional screening and treatment (such as cervical length screening, bacterial vaginosis screening, administration of progesterone, and when indicated a cerclage (Guideline: Prevention of recurrent spontaneous preterm birth. Dutch Soc Obs Gynaecol. 2024;(v 2.0)). |
| C. Changes made | None |
| D. Location of changes | N.A. |

Reviewer 4; Comment 6

| A. Reviewer | The anticipated adverse outcome was anticipated to be 12% in pessary, and it was ~19% in the trial, and the anticipated adverse outcome was anticipated to be 24% in progesterone, and it was ~13%. This is supremely confusing. Why? The authors attempt to address this in lines 374-390 but it is unfulfilling as they conclude if they had not stopped for futility perhaps, they would be able to provide more clarity.  Please provide more information on why the decision to halt was made with only 56 patients left to recruit - without the planned recruitment it leaves so many questions unanswered. |
| --- | --- |
| B. Response | Thank you for this important comment, this is a highly pertinent issue—one with which we, as a research group, also struggled significantly, as mentioned earlier in comment 2 of reviewer 2.  As outlined in the manuscript, interim analyses were conducted by an independent Data and Safety Monitoring Committee (DSMC). When the DSMC’s recommendation to halt the study because of futility (not reaching superiority of pessary) was communicated to us, we formally raised objections; however, the board maintained its position. We subsequently referred the matter to the Medical Ethics Review Committee (METC) of the Amsterdam UMC, where an independent panel reviewed the case. Despite the initial agreement that the study would not be discontinued on grounds of futility alone, the committee concluded that the DSMC’s recommendation should be upheld. As a result, the study was terminated prematurely.  Superiority of pessary treatment could not be demonstrated; however, it remains unclear whether a significant benefit of progesterone could have been detected. So, answering the question why remains impossible other than we have done. |
| C. changes made | The following explanation is added to the results section:  “Although no formal futility stopping rules based on the statistical significance of the effect of the treatment were prespecified in the statistical analysis plan, the DSMC considered continued recruitment scientifically unjustified based on the observed data. Following an independent review by the METC of the Amsterdam UMC, it was concluded that the DSMC’s advice should be followed, leading to early termination of the trial.” |
| D. Location of changes | Lines 331-335 |

Reviewer 4; Comment 7

| A. Reviewer | The conclusion is the authors did not find superiority of pessary for management over progesterone, indeed the pessary performed quite poorly in this trial.  How many discontinued the pessary?  How many discontinued the vaginal progesterone |
| --- | --- |
| B. Response | We would like to refer to Table S1  53 women discontinued the protocol in the pessary group and 41 women in the progesterone group. |
| C. changes made | None |
| D. Location of changes | N.A. |

**General editorial comments**

Editorial Comment 1

| A. Reviewer | It is not made sufficiently clear in the main text that the trial did not reach its planned sample size i.e. that it was underpowered. Please comment on this detail in the main manuscript text. |
| --- | --- |
| B. Response | Thank you for pointing out this omission; as mentioned in earlier comments (reviewer 4, comment 6 and reviewer 2, comment 2), we have added and explanation to the results section. |
| C. Changes made | The following explanation is added to the results section:  “Although no formal futility stopping rules based on the statistical significance of the effect of the treatment were prespecified in the statistical analysis plan, the DSMC considered continued recruitment scientifically unjustified based on the observed data. Following an independent review by the METC of the Amsterdam UMC, it was concluded that the DSMC’s advice should be followed, leading to early termination of the trial.” |
| D. Location of changes | Lines 331-335 |

Editorial Comment 2

| A. Reviewer | Inclusion/exclusion criteria: Death of one or both of the foetuses in this pregnancy not explicitly specified in the main manuscript text. |
| --- | --- |
| B. Response | Thank you for pointing this out. We have added the exclusion criteria. |
| C. Changes made | Addition of: “.., death of one or both of the foetuses in this pregnancy,” |
| D. Location of changes | Lines 187-188 |

Editorial Comment 3

| A. Reviewer | The study protocol should include details of any amendments, as well as the date of their approval by the institutional review or ethics committee. Please also detail any deviations from the study protocol in the Methods section of your manuscript. |
| --- | --- |
| B. Response | We were not previously aware that this information was missing from the manuscript. We have now added a reference to Supplementary Table S4 in the Methods section, where all protocol amendments, their dates of ethical approval, and deviations from the study protocol are detailed. |
| C. Changes made | We have added the following information to the methods section:  “Details of all protocol amendments, including dates of approval by the institutional ethics committee, as well as any deviations from the study protocol, are provided in Supplementary Table S1.” |
| D. Location of changes | Lines 178-180 |

Editorial Comment 4

| A. Reviewer | Likewise, please confirm that the statistical analysis plan provided (version 2.0, dated 5th June 2024) is the final version of this document prior to data lock. Please note that version number and date of the statistical analysis plan differ on page 1 of the pdf; please clarify. |
| --- | --- |
| B. Response | Yes, we confirm that the Statistical Analysis Plan (SAP) version 2.0, dated 5th June 2024, is the final version of the document prior to data lock. The differing date on page 1 of the PDF (stating 2025 instead of 2024) was a typographical error, for which we sincerely apologize. Thank you for bringing this to our attention. |
| C. Changes made | We have corrected the date of the SAP final version on page 1. |
| D. Location of changes | N.A. |

Editorial Comment 5

| A. Reviewer | From protocol version 3.0 (dated 17th April 2020) and the statistical analysis plan (version 2.0, dated 5th June 2024) we note the following:  - Secondary outcomes reported but not prespecified in the protocol:  PTB before 24 weeks  Premature rupture of the membranes  Mode of delivery  Placed cerclages  Birth weight  Individual components of the composite neonatal outcome Patent ductus arteriosus (PDA) Neonatal seizures Vaginal blood loss Excessive vaginal discharge Twin-to-twin Transfusion Syndrome  - Secondary outcomes pre-specified in the protocol but not reported:  Admission days for preterm labour  Costs  Please clarify and explain all discrepancies between the paper and protocol. If the outcomes were not prespecified in the protocol, please define them in the Methods (Outcomes section) as post hoc and explain why they were added. Post-hoc comparisons should be presented as hypothesis generating rather than conclusive.  Please ensure that all prespecified outcomes (primary, secondary, and exploratory) are listed in the Methods/Outcomes section and indicate whether there are outcomes that are not presented in the current report. |
| --- | --- |
| B. Response | With respect to the secondary outcomes that were reported in the manuscript but not explicitly pre-specified in the protocol, we would like to clarify that these outcomes reflect baseline characteristics and clinically relevant outcomes that are part of the expected description of a pregnant population at the time of delivery. These were comprehensively defined and documented in the Statistical Analysis Plan (SAP, version 2.0, dated 5 June 2024), which served as our guiding document for finalizing all analyses prior to conducting them. Therefore, these are not post hoc outcomes; they were planned and agreed upon in advance. The fact that some of these were not explicitly listed in the original protocol likely reflects a lack of precision in the early phase of protocol drafting, rather than a retrospective addition of outcomes.  Regarding the secondary outcomes that were pre-specified in the protocol but not reported in the manuscript (namely, admission days for preterm labour and cost outcomes), we chose to address these in a separate cost-effectiveness analysis (CEA). This decision was made to ensure clarity and methodological consistency across analyses, with the relevant outcomes to be presented in that dedicated report.  We trust this clarifies the rationale behind the reporting decisions and the alignment between our protocol, SAP, and manuscript. |
| C. Changes made | We have added the explanation why we have not address the admission days and cost outcomes: “Regarding the secondary outcomes that were pre-specified in the protocol but not reported in the manuscript (namely, admission days for preterm labour and cost outcomes), we chose to address these in a separate cost-effectiveness analysis (CEA).” |
| D. Location of changes | Lines 254-256 |
