# Supplementary material for: Cervical pessary versus vaginal progesterone in women with a multiple pregnancy and a short cervix: A randomised controlled trial
Source: PLoS Med. 2025 Nov 3;22(11):e1004586. doi: 10.1371/journal.pmed.1004586 (PMC12591417; doi:10.1371/journal.pmed.1004586)
Supplement: Attachment — Submitted filename: QP_Multiples_Letter of Response_resubmission2_final.docx [file pmed.1004586.s010.docx]

***Suzanne de Bruijn, PhD***

***Associate Editor***

***PLOS Medicine***

Date: 26-sep-25

Our reference: PMEDICINE-D-25-00762R1

Title: **Cervical pessary versus vaginal progesterone in women with a multiple pregnancy and a short cervix: a cluster randomised controlled trial**

**Subject: Re-submission of manuscript**

Dear Suzanne De Bruijn,

We sincerely thank you, the editors, and the reviewers for the thoughtful assessment of our resubmitted manuscript and for the constructive guidance throughout the review process. We are pleased to hear that the manuscript is now considered suitable for publication in *PLOS Medicine* pending the resolution of the remaining editorial and production issues. We have addressed the reviewers’ points in our response letter and revised the manuscript accordingly. The editorial requests have also been implemented as detailed in the accompanying point-by-point response.

With regard to data availability, we have revised our statement to clarify that, due to the sensitive and identifiable nature of the participant data, these cannot be made publicly available. Instead, de-identified data are available upon reasonable request via an independent committee, as described in the revised Data Availability Statement.

We appreciate your consideration and look forward to the next steps in the review process.

Sincerely,

Charlotte van Dijk, on behalf of all co-authors,

Department of Obstetrics and Gynaecology

Amsterdam UMC, Amsterdam,

The Netherlands

**Comments from Reviewers:**

Reviewer 2; comment 1

| A. Reviewer | **I thank the authors for their considered responses to my comments. However, I ask the authors to reconsider two of my previous comments; I do not feel that these have been adequately addressed. I had previously stated that this is an example of a cluster randomised trial (since pregnant women were randomised and outcomes measured on their babies, and mothers had multiple babies) - I stand by this. This is an example of a trial where some clusters were of size 2, and some of size 3; if outcomes were measured only on mothers (i.e. if there were no infant-level outcomes) then I would agree that this is not a cluster randomised trial. However, since the primary outcome is measured on babies, this is classic example of a cluster randomised trial. Please see this paper on accounting for twins in sample size calculations (PMID: 29727020). (This may be useful for any future studies the investigators plan to conduct in a similar population - it indicates how twins may be accounted for). I also note that if the within-mother correlation was not accounted for then this study was likely underpowered to detect the effect of interest (nothing can be done about this now, but it should be noted).** |
| --- | --- |
| B. Editorial response | We discussed the issue of this being a clustered RCT with the academic editor, who stated "technically the trial is clustered as the infants are in the outcomes, but practically it would be more confusing to call it a cluster trial". |
| C. Authors response | Thank you for carefully revisiting this important methodological point and for providing a useful reference. We appreciate the argument that our trial could technically be regarded as a cluster randomized design. However, following editorial guidance, we have retained the presentation of the trial as an individually randomized design. We believe that the correction for clustering now applied in the analyses adequately addresses this issue, while we also acknowledge that accounting for clustering from the outset would be advisable in future trials in similar populations. We further note the reviewer’s important comment regarding the potential for underpowering, which we have mentioned in the discussion. |
| D. Changes made | Addition of the following limitation: Another limitation concerts its design. While at the time of protocol development and in previous similar studies the trial was not considered as a cluster randomized design, we have corrected for clustering in the current analyses, which we consider sufficient; nevertheless, we acknowledge that in future studies such clustering should be addressed from the outset, and that the lack of initial adjustment may have led to some degree of underpowering. |
| E. Location of changes | Lines 467-471 |

Reviewer 2; comment 2

| A. Reviewer | **I continue to disagree with the per-protocol analysis as described, and continue to recommend alternative approaches (e.g. estimation of complier average causal effects as stated in my previous comment). If the authors do not wish to perform this analysis and instead include the per protocol analysis, the limitations of this must be clearly stated - i.e. the result observed may be due to confounding (those who adhered may be very different from those who did not) and thus is difficult to interpret.** |
| --- | --- |
| B. Editorial response | We appreciate the 2nd comment from Reviewer #2, but we think the prespecified analysis needs to be reported on. |
| C. Authors resonse | Thank you again highlighting important considerations regarding the per-protocol analysis. We fully recognize the limitations of this approach, particularly the potential for confounding and the consequent challenges in interpretation, as the reviewer has noted. However, as this analysis was prespecified in our protocol, we believe it is important to report it transparently. In line with the reviewer’s recommendation, we have now more explicitly stated the limitations of the per-protocol analysis in the discussion section. |
| D. Changes made | However, per-protocol analyses are inherently prone to confounding, as women who adhered may differ systematically from those who did not, and the findings should therefore be interpreted with caution. |
| E. Location of changes | Lines 465-467 |

Reviewer 2; comment 3

| A. Reviewer | **Instead of stating "and continuous outcomes were analysed using GEE with a linear distribution and identity link with mean differences and the corresponding 95% confidence intervals along with the p-value." It would be more appropriate to state "and continuous outcomes were analysed using GEE with an identity link with mean differences and the corresponding 95% confidence intervals along with the p-value reported."** |
| --- | --- |
| B. Response | We adjusted the description of the analysis. |
| C. Changes made | Removal of ‘linear distribution and’ |
| D. Location of changes | Line 300 |

Reviewer 4, comment 1

| A. Reviewer | **The authors have extensively addressed all comments raised by the reviewers and editors and have modified the manuscript accordingly. The manuscript is improved with these additions.** |
| --- | --- |
| B. Response | We thank the reviewer for their positive feedback and are pleased that the revisions have improved the manuscript. |
| C. Changes made | NA |
| D. Location of changes | NA |

**General editorial requests**

* Please confirm that your abstract complies with our requirements, including format (three sections: Background, Methods and Findings, and Conclusions) and providing all the information relevant to this study type ([link](https://eur04.safelinks.protection.outlook.com/?url=https%3A%2F%2Fjournals.plos.org%2Fplosmedicine%2Fs%2Fsubmission-guidelines%23loc-abstract&data=05%7C02%7Cc.e.vandijk%40amsterdamumc.nl%7C90a61a3141ce431e40f208ddf76653d5%7C68dfab1a11bb4cc6beb528d756984fb6%7C0%7C0%7C638938741045946049%7CUnknown%7CTWFpbGZsb3d8eyJFbXB0eU1hcGkiOnRydWUsIlYiOiIwLjAuMDAwMCIsIlAiOiJXaW4zMiIsIkFOIjoiTWFpbCIsIldUIjoyfQ%3D%3D%7C0%7C%7C%7C&sdata=c0apcih5Ac7Yof1dgS1vXn7addIex0l9nbZeAOx24GU%3D&reserved=0))

*=> yes we confirm*

* Please ensure that all abbreviations are defined at first use throughout the text

*=> check*

* Please confirm that all numbers presented in the abstract are present and identical to numbers presented in the main manuscript text.

*=> yes we confirm*

* We appreciate the inclusion of the files for the study approval, as well as supplement F "VOC declaration of intent regarding incubator infants.pdf" ; However, could you change the files in our system from 'supplement' to 'supporting', as otherwise they will get published with the paper.

*=> We have adjusted the files to supporting instead of supplement.*

**Other editorial requests**

GENERAL

* Please review your text for claims of novelty or primacy (e.g. 'for the first time') and remove this language. In addition, please check that any use of statistical terms (such as trend or significant) are supported by the data, and if not please remove them.

*=> We reviewed and adjusted the text where seems appropriate for both the claims of novelty/primacy and statistical terms.*

* Please remove the 'conclusions' subheading from the discussion. Please also remove any other subheadings from the discussion.

*=> We have removed the subheadings.*

* Statistical reporting: Please revise throughout the manuscript, including tables and figures.

- Please report statistical information as follows to improve clarity for the reader ""22% (95% CI [13,28]; p</=)"".

- Please separate upper and lower bounds with commas instead of hyphens as the latter can be confused with reporting of negative values.

- Please repeat statistical definitions (HR, CI etc.) for each set of parentheses.

*=> We revised the statistical reporting wherever necessary.*

-Author summary: Please include that the trial was halted for futility.

*=> We stated in the Author Summary that the trial was halted for futility.*

FUNDING STATEMENT

* The funding statement should include: specific grant numbers, initials of authors who received each award, URLs to sponsors’ websites. Also, please state whether any sponsors or funders (other than the named authors) played any role in study design, data collection and analysis, the decision to publish, or preparation of the manuscript. If they had no role in the research, include this sentence: “The funders had no role in study design, data collection and analysis, decision to publish, or preparation of the manuscript.”

* It appears that one or more study authors is affiliated with one or more of the agencies that funded the study. Thus, the statement “The funders had no role in study design, data collection and analysis, decision to publish, or preparation of the manuscript” does not apply. Please revise the Financial Disclosure accordingly, as in "[Author name] is [author's role] at [funding agency]. The funders had no other role in study design…..”

*=> We have adjusted the funding/financial disclosure statement.*

COMPETING INTERESTS STATEMENT

* All authors must declare their relevant competing interests per the PLOS policy, which can be seen here[: link.](https://eur04.safelinks.protection.outlook.com/?url=https%3A%2F%2Fjournals.plos.org%2Fplosmedicine%2Fs%2Fcompeting-interests&data=05%7C02%7Cc.e.vandijk%40amsterdamumc.nl%7C90a61a3141ce431e40f208ddf76653d5%7C68dfab1a11bb4cc6beb528d756984fb6%7C0%7C0%7C638938741045960453%7CUnknown%7CTWFpbGZsb3d8eyJFbXB0eU1hcGkiOnRydWUsIlYiOiIwLjAuMDAwMCIsIlAiOiJXaW4zMiIsIkFOIjoiTWFpbCIsIldUIjoyfQ%3D%3D%7C0%7C%7C%7C&sdata=p3ojDenDDAP7vXKlKud%2Bh9xoqoclIw%2BHb0c11iiuJu4%3D&reserved=0)

For authors with ties to industry, please indicate whether any of the interests has a financial stake in the results of the current study.

*=> We have collected and submitted all competing interests forms of all the authors and stated their competing interests in the manuscript.*

DATA AVAILABILITY

* PLOS Medicine requires that the de-identified data underlying the specific results in a published article be made available, without restrictions on access, in a public repository or as Supporting Information at the time of article publication, provided it is legal and ethical to do so. Please see the [policy](https://eur04.safelinks.protection.outlook.com/?url=http%3A%2F%2Fjournals.plos.org%2Fplosmedicine%2Fs%2Fdata-availability&data=05%7C02%7Cc.e.vandijk%40amsterdamumc.nl%7C90a61a3141ce431e40f208ddf76653d5%7C68dfab1a11bb4cc6beb528d756984fb6%7C0%7C0%7C638938741045974437%7CUnknown%7CTWFpbGZsb3d8eyJFbXB0eU1hcGkiOnRydWUsIlYiOiIwLjAuMDAwMCIsIlAiOiJXaW4zMiIsIkFOIjoiTWFpbCIsIldUIjoyfQ%3D%3D%7C0%7C%7C%7C&sdata=9PBmTvy6ivgPCpZA9fR8E2qubseY43En6B9sdNciwcw%3D&reserved=0) at and [FAQs](https://eur04.safelinks.protection.outlook.com/?url=http%3A%2F%2Fjournals.plos.org%2Fplosmedicine%2Fs%2Fdata-availability%23loc-faqs-for-data-policy&data=05%7C02%7Cc.e.vandijk%40amsterdamumc.nl%7C90a61a3141ce431e40f208ddf76653d5%7C68dfab1a11bb4cc6beb528d756984fb6%7C0%7C0%7C638938741045994122%7CUnknown%7CTWFpbGZsb3d8eyJFbXB0eU1hcGkiOnRydWUsIlYiOiIwLjAuMDAwMCIsIlAiOiJXaW4zMiIsIkFOIjoiTWFpbCIsIldUIjoyfQ%3D%3D%7C0%7C%7C%7C&sdata=Fu53XJrWIMF1As3k7i6B9bzJ%2B6DEbZcl%2BICtQeexJrk%3D&reserved=0%22).

* The Data Availability Statement (DAS) requires revision. For each data source used in your study:

a) If the data are freely or publicly available, note this and state the location of the data: within the paper, in Supporting Information files, or in a public repository (include the DOI or accession number).

b) If the data are owned by a third party but freely available upon request, please note this and state the owner of the data set and contact information for data requests (web or email address). Note that a study author cannot be the contact person for the data.

c) If the data are not freely available, please describe briefly the ethical, legal, or contractual restriction that prevents you from sharing it. Please also include an appropriate contact (web or email address) for inquiries (again, this cannot be a study author).

*=> We adjusted the Data Availability statement .*

FIGURES AND TABLES

* Please provide titles and legends for all figures and tables (including those in Supporting Information files). Please define all acronyms used in each figure or table in its corresponding legend. For Table 2, the legend seems to be part of the table.

*=> All figures and tables are provided with titles and legends and used acronyms are defined.*

* When a p value is given, please specify the statistical test used to determine it in the legend.

*=> statistical tests are included in the legends.*

CLINICAL TRIALS

* Some of the outcome measures or methods appear to differ between the submitted manuscript and the trial registry and/or protocol. Please clarify and explain the discrepancy. If the outcomes were not prespecified in the protocol, please indicate that they were post hoc and explain why they were added. Post hoc comparisons should be presented as hypothesis generating rather than conclusive.

*=> We have specified which outcomes we have added to the statistical analysis plan, since they were not mentioned in the original protocol.*

*See lines 262-266.*
